# Preparation of Acidic 5-Hydroxy-1,2,3-triazoles via the Cycloaddition of Aryl Azides with β-Ketoesters

**DOI:** 10.1021/acs.joc.1c00778

**Published:** 2021-07-27

**Authors:** Roberta Pacifico, Dario Destro, Malachi W. Gillick-Healy, Brian G. Kelly, Mauro F. A. Adamo

**Affiliations:** † Centre for Synthesis and Chemical Biology (CSCB), Department of Chemistry, Royal College of Surgeons in Ireland, 123 St. Stephen’s Green, Dublin 2, Ireland; ‡ KelAda Pharmachem Ltd., A1.01 Science Centre South, Belfield, Dublin 4, Ireland

## Abstract

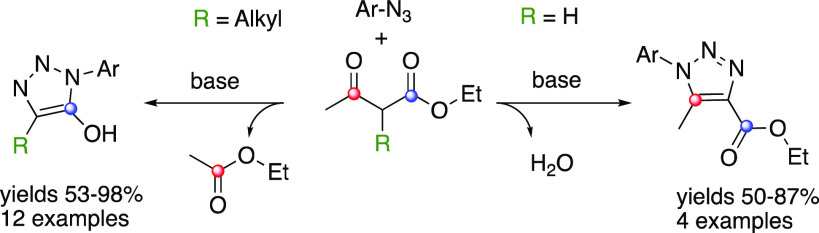

Herein, a high-yielding cycloaddition reaction of β-ketoesters and azides to provide 1,2,3-triazoles is described. The reactions employing 2-unsubstituted β-ketoesters were found to provide 5-methyl-1,2,3-triazoles, whereas 2-alkyl-substituted β-ketoesters provided 5-hydroxy-1,2,3-triazoles (shown to be relatively acidic) in high yields and as single regioisomers. Several novel compounds were reported and characterized including long-chain 5-hydroxy-1,2,3-triazoles potentially bioisosteric to hydroxamic acids.

## Introduction

**Figure 1:**
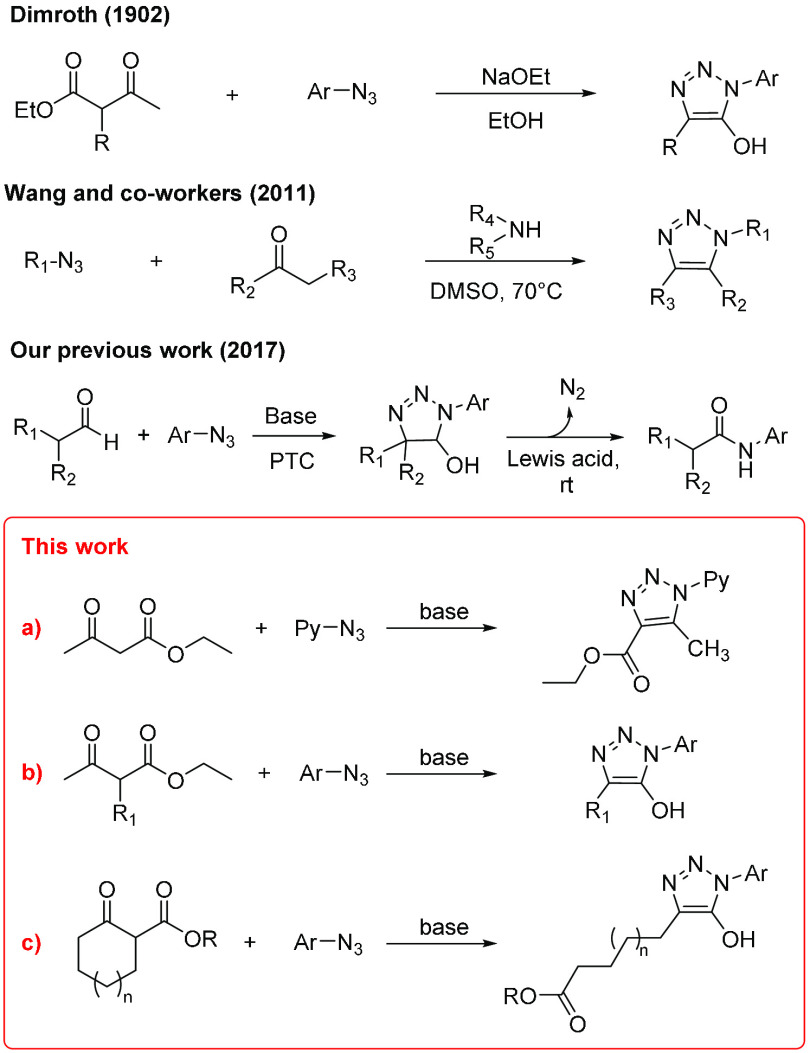
Historical and novel approaches for the synthesis of 1,2,3-triazoles.

1,2,3Ttriazoles are important scaffolds employed in medicinal chemistry,^1^ catalysis,^2^ materialscience,^3^ and biology.^4^ The electronic and physicochemical properties of triazoles bear a close similarity to those of the amide functionality and therefore can be classified as amide bioisosteres.^5,6^ Copper-catalyzed alkyne–azide cycloaddition is the most accredited method to synthesize substituted 1,2,3-triazoles,^7−11^ while the less well-known reactions of malonates or β-ketoesters with aromatic azides have become attractive alternatives as they do not require metal catalysts to proceed (Figure 1).^12^

β-Ketoesters react quickly with mild bases, providing highly reactive enolates that have a myriad of reported applications in organic chemistry.^13,14^ Dimroth reported a cycloaddition of β-ketoesters and azides in 1902^15^ where ethyl acetoacetate reacted with azidobenzene in the presence of sodium ethoxide to provide 5-hydroxytriazoles in poor yields. This material was properly identified; however, it was not characterized beyond the physical constants and, notably, their remarkable acidity was overlooked. More recently, Wang and co-workers reported the cycloaddition of β-ketoesters and azides, via organo-catalysis, to yield 1,4-disubstituted 1,2,3-triazoles.^16^ Intrigued by these reports^15,16^ where the same reagents gave rise to different products under similar basic conditions, and in a continuation of our studies on the reactivity of azides and enolates (Figure 1),^17^ we decided to re-examine the cycloaddition of simple ketoesters and aromatic azides in the presence of organic bases.

## Results and Discussion

This work involved reacting a selection of pyridyl- or aryl-azides and β-ketoesters and showed that distinct triazole products could be obtained in high yields via a divergent reaction pathway that led to either disubstituted 1,2,3-triazoles or 5-hydroxy-1,2,3-triazoles depending upon the nature of the β-ketoester. These findings were subsequently exploited to prepare a family of 5-hydroxytriazoles, whose acidity has been shown to be even stronger (p*K*_a_ = 4.20 for **5a**) than those for analogous compounds (p*K*_a_ = 5.14–6.21) reported by Sainas and co-workers.^18^ Our observation of the relatively strong acidity of 5-hydroxytriazoles has the potential to be applied toward hydroxamic acid bioisosteres (Figure 1).

Our investigation began with the cycloaddition reaction of pyridyl azides **2a**–**c** with ethyl acetoacetate **1a** in the presence of organic soluble bases, from which 1,8-diazabicyclo[5.4.0]undec-7-ene (DBU) gave the best yield. The reactions of pyridyl azides **2a** and **2b** with **1a** furnished triazoles **3a** and **3b** in 80% and 72% isolated yields, respectively, whereas the reaction of pyridyl azide **2c**, bearing an *ortho*-nitrogen, showed no conversion to **3c** (Table 1).

**Table 1:**
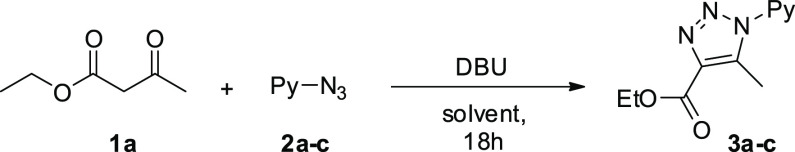
Cycloaddition of β-Ketoester **1a** with Aryl Azides **2**^a^

entry	keto ester	aryl azide	Py	product	catalyst	solvent	yield (%)^b^
1	**1a**	**2a**	4-pyridyl	**3a**		MeCN	80
2	**1a**	**2b**	3-pyridyl	**3b**		MeCN	72
3	**1a**	**2c**	2-pyridyl	**3c**		MeCN	0
4	**1a**	**2c**	2-pyridyl	**3c**	Cu(OTf)_2_·C_6_H_5_CH_3_	DMSO	50

a Reaction conditions are as follows: **1a** (1.2 equiv), **2a**–**c** (1 equiv), DBU (1.2 equiv), and solvent (0.2 M).

b Isolated yield.

**Figure 2:**
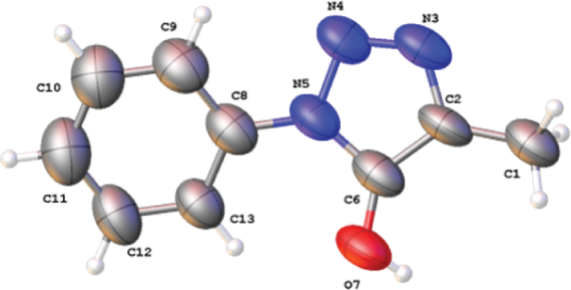
X-ray crystal structure of 5-hydroxytriazole 5a.^27^

Compound **3c**, however, was obtained in average yields (50%) when a copper trifluoromethanesulfonate toluene complex (Cu(OTf)_2_·C_6_H_5_CH_3_) in dimethyl sulfoxide (DMSO) was added to the reaction mixture. The reaction of **1a** and phenyl azide **4a** (Table 2, entry 1) gave the disubstituted triazole ester **3d**(16) in an excellent isolated yield. In summary, the reaction of unsubstituted **1a** with azides **2a**–**c** (Table 1) or **4a** (Table 2) under basic conditions provided 4,5-disubstituted 1,2,3-triazoles of a similar type to those reported by Wang.^16^ However, when 2-substituted β-ketoester **1b** was reacted with azide **4a** (Table 2, entry 2), a sharp diversion of the reaction course was observed, and 5-hydroxy-triazole **5a** was isolated as the sole product (Table 2 entry 2). We then investigated the scope of the reaction, applying the same reaction protocol to β-ketoesters **1b** and **1c** and aryl azides (**4a**–**j**) (Table 2). Reactions of ketoester **1b** with aryl azides **4h**–**j** did not lead to the formation of any product, and only starting materials were recovered after 18 h. Reactions of ketoesters **1b** and **1c** with azides **4a**–**g** and **4a** under identical conditions provided 5-hydroxytriazoles **5a**–**g** and **5k**, which with the exception of **5a** are reported here for the first time. The chemical structure of **5a** was confirmed by single-crystal X-ray diffractometry (see Figure 2 and the Supporting Information).

**Table 2:**
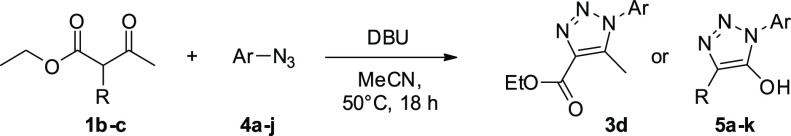
Cycloaddition of β-Ketoesters and Aryl Azides to Give 5-Hydroxytriazoles^a^

entry	ketoester	R	aryl azide	Ar	product	time (h)	yield (%)^b^
1	**1a**	H	**4a**	Ph	**3d**	18	87
2	**1b**	Me	**4a**	Ph	**5a**	6	80
3	**1b**	Me	**4a**	Ph	**5a**	18	98^c^
4	**1b**	Me	**4b**	4-BrC_6_H_4_	**5b**	18	87
5	**1b**	Me	**4c**	4-OMeC_6_H_4_	**5c**	18	62
6	**1b**	Me	**4d**	2-PhC_6_H_4_	**5d**	18	75
7	**1b**	Me	**4e**	1-naphthyl	**5e**	18	79
8	**1b**	Me	**4f**	4-NO_2_C_6_H_4_	**5f**	18	53
9	**1b**	Me	**4g**	2-NO_2_-4-MeC_6_H_3_	**5g**	18	56
10	**1b**	Me	**4h**	2-Br-C_6_H_4_	**5h**	18	n.r.^d^
11	**1b**	Me	**4i**	*n*-Bu	**5i**	18	n.r.^d^
12	**1b**	Me	**4j**	Bn	**5j**	18	n.r.^d^
13	**1c**	Bn	**4a**	Ph	**5k**	18	75

a Reaction conditions are as follows: **1b** or **1c** (1.2 equiv), **4a**–**j** (1 equiv), and DBU (1.2 equiv) in MeCN (0.2 M) at 50 °C.

b Isolated yield.

c ^1^H-NMR spectroscopic yield.

d No reaction.

The reaction of ketoesters **1b** and **1c** with aryl azides **4a**–**j** in the presence of 1.2 equiv of DBU in acetonitrile at 50 °C for 18 h provided 5-hydroxytriazoles **5a**–**k** in moderate to good yields. The reaction was, however, sensitive to the electronic effects of substituents of the azide, where electron-neutral azides **4a**, **4b**, **4d**, and **4e** gave highest yields. The electron-rich azide **4c** reacted well, albeit the yields were lower. Aromatic azides **4f**–**g** bearing strong electron-withdrawing groups provided the corresponding 5-hydroxytriazoles **5f**–**g** in only moderate yields. Steric hindrance from aryl substituents of aryl azides **4** was not found to affect reaction yield. The potential for the organo-catalytic activity of DBU in this reaction was also investigated; however, no evidence to support this was found. We then proceeded to optimize the reaction conditions between **1b** and **4a** by varying the base, solvent, temperature, and reaction times.

The high yield obtained when reacting **1b** with **4a** in the presence of solid KOH (potassium hydroxide) as a base and TBAB (tetrabutylammonium bromide) as a PTC (phase-transfer catalyst) encouraged us to study the scope of this transformation (Table 3). Phase-transfer conditions were found to be particularly effective with electron-poor and electron-neutral azides, such as **4a**, **4g**, and **4h**, with yields up to 95%. However, when bulky azide substituents where present, such as **4e**, only the hydrolyzed product **6** was favored over cyclization (Table 3). Since compound **5a** behaved as a relatively strong Brønsted acid, we decided to carry out a titration experiment to better characterize its properties. The p*K*_a_ of **5a** was found to be 4.2, which is comparable to that of a carboxylic acid (see the Supporting Information).^19^ This result was very unexpected compared to the p*K*_a_ values of the related 4-hydroxy-1,2,3-triazoles, which were found to be significantly less acidic than those reported by Pippione and co-workers.^20^ Moreover, we found 5-hydroxytriazoles to be highly soluble in water when accompanied by both organic and inorganic bases, thus justifying their attractiveness as candidates for drug discovery. We further expanded the scope of the cyclization reaction to include cyclic β-ketoesters **7a**–**c** to provide novel long-chain 5-hydroxytriaxoles **8**. Interestingly, poor conversion was observed when the reaction was performed in solvent, while we saw a notable improvement when the cyclization was performed solvent-free with yields up to 79% (Table 4).

**Table 3:**
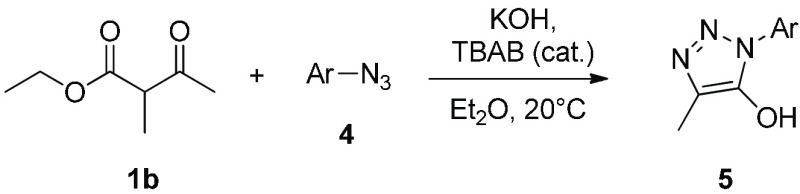
Synthesis of 5-Hydroxytriazoles: PTC-Mediated Cycloaddition of β-Ketoester **1b** and Aryl Azides **4**^a^

entry	aryl azide	Ar	product	time (h)	yield (%)^b^
1	**4a**	Ph	**5a**	4	95
2	**4e**	1-naphthyl	**5e**	18	n.r.^c^
3	**4g**	4-NO_2_C_6_H_4_	**5f**	4	76
4	**4h**	2-NO_2_-4-MeC_6_H_3_	**5g**	4	83

a Reaction conditions are as follows: **1b** (1.2 equiv), **4** (1.2 equiv), TBAB (0.1 equiv), and KOH (2.2 equiv) in Et_2_O (0.2 M) at 20 °C.

b Isolated yield.

c Only the hydrolysis product 2-methyl-3-oxobutanoic acid (**6**) was formed.

**Table 4:**
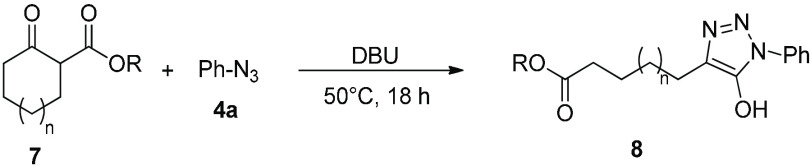
Synthesis of Long-Chain 5-Hydroxytriazoles^a^

entry	ketoester	R	*n*	solvent	product	yield (%)^b^
1	**7a**	Et	1	MeCN	**8a**	29
2	**7a**	Et	1	none	**8a**	44
3	**7b**	Me	2	none	**8b**	79
4	**7c**	Et	3	none	**8c**	38

a Reaction conditions are as follows: **7** (1.2 equiv), **4a** (1 equiv), and DBU (1.2 equiv) at 50 °C for 18 h.

b Isolated yield.

**Figure 3:**
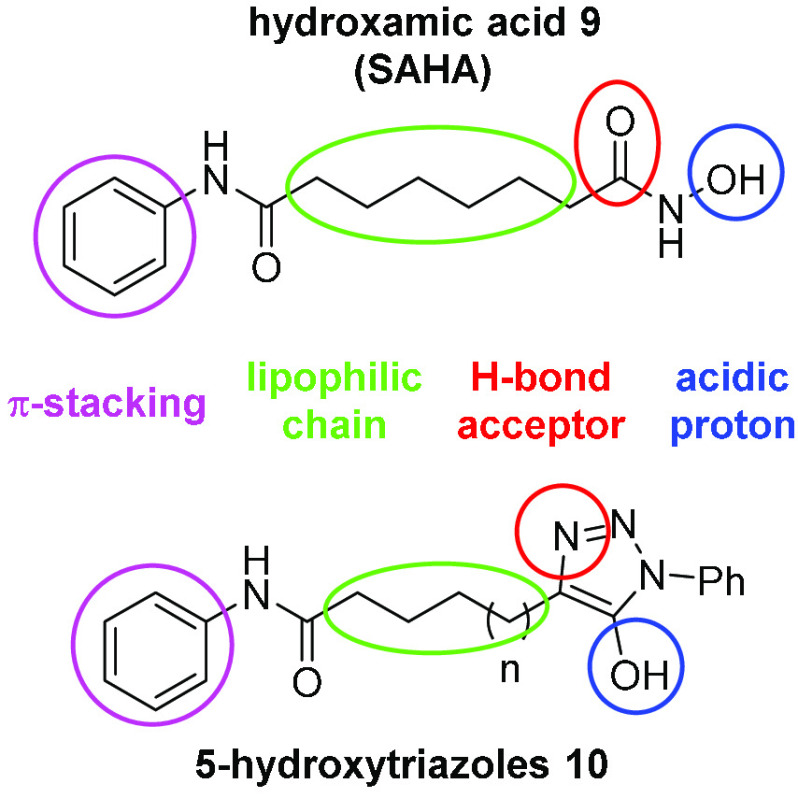
Structural similarities between HDAC inhibitor SAHA **(**9) and long-chain 5-hydroxytriazoles (**10a**–**c**).

A literature search highlighted the structural similarity of 5-hydroxytriazoles and hydroxamic acids.^21^ Hydroxamic acids and 5-hydroxytriazoles share similar p*K*_a_ valuesand amide-like bioisosterism, which makes them excellent ligands for enzyme-bound metals. Compounds **5a**–**g** and **8** reacted with Fe^2+^ and Cu^2+^ salts to provide blue-violet and red-colored solutions, indicating a ligand-like behavior similar to that of hydroxamic acids. Suberanilohydroxamic acid **9** (SAHA) is a hydroxamic acid that is active as a histone deacetylase (HDAC) inhibitor.^22,23^ To exemplify the potential of the 5-hydroxytriazole nucleus in medicinal chemistry, we set out to convert long-chain 5-hydroxytriazoles **8a**–**c** into terminal *N*-phenylamide-substituted triazoles **10a**–**c**, which bear a close structural relationship to hydroxamic acid **9** (Figure 3).

The preparation of **10a**–**c** (*n* = 1–3, respectively) is reported in the Experimental section. The structural and functional similarities between hydroxamic acid **9** and 5-hydroxytriazoles **10** are highlighted in Figure 3 and include the following: (i) nominally similar scaffolds and chemical functionalities; (ii) analogous lone pairs (circled in red) of the carbonyl oxygen of the hydroxamic acid and the pyridyl-like N of the triazole system;^24^ (iii) hydrophobic backbones (circled in green), which are essential for interaction with active sites of HDAC isoforms, e.g., zinc-binding groups; and (iv) aromatic rings (in pink), which are essential for the correct positioning in the enzyme active site via π–π stacking.^25^

**Scheme 1:**
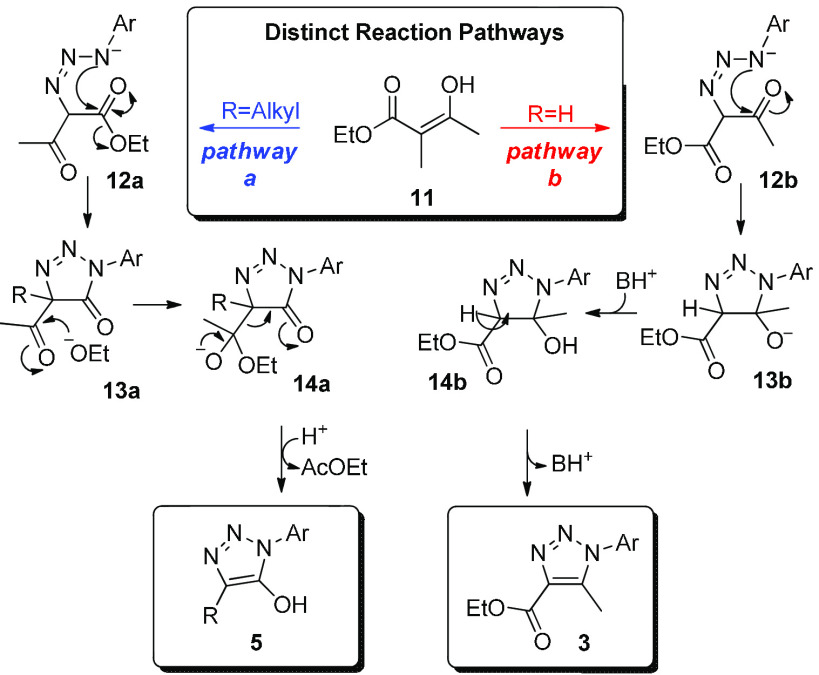
Proposed Distinct Mechanistic Pathways for the Formation of 5-Hydroxytriazoles **5** (Pathway *a*) versus 5-Methyl-triazoles **3** (Pathway *b*)

Based on the reactivity observed, two reaction mechanisms have been proposed (Scheme 1), which lead to distinct products via analogous intermediates **12a** and **12b**. Intermediates **12a** and **12b** arise from reaction of enolate **11** with aryl azides (pathway *a* or pathway *b*), respectively. In pathway a, species **12a** is formed and will evolve toward cyclic amide **13a**, which contains no enolizable proton, and the concomitant elimination of ethoxide. A subsequent attack of ethoxide to the acetoxy group in **13a** will lead to the elimination of ethyl acetate and the formation of **5**. Conversely, in the presence of an enolizable proton such as in **12b** (pathway *b*), the following cyclization to **13b** and its subsequent protonation will generate **14b**, which will provide compounds **3** after dehydration. A rationale similar to ours used to explain the mechanism in Scheme 1 has been reported by Pedersen and Begtrup for the reaction between phenyl azides and amides of malonic acids.^26^

## Conclusion

In conclusion, we have demonstrated that aryl azides undergo two distinct cycloaddition reactions with enolizable β-ketoesters depending on the substitution pattern on the β-ketoester, leading to different products. The cycloaddition of 2-unsubstituted β-ketoester with pyridyl azides and phenyl azide was found to lead to 5-methyl-triazoles, whereas the cycloaddition of 2-alkyl-substituted β-ketoesters with phenyl azide and substituted aryl azides was found to lead to 5-hydroxytriazoles, where the structure of one of the products has been confirmed via X-ray diffractometry (see Figure 2 and the Supporting Information).^27^ The reaction of phenyl azide and substituted aryl azides with 2-alkyl-substituted β-ketoesters has been shown to be a fast, mild, and high-yielding method for the synthesis of 5-hydroxy-1,2,3-triazoles. The relatively acidic nature we observed for the 5-hydroxytriazoles has led us propose the study of 5-hydroxytriazole analogues as a new class of bioisosteres of hydroxamic acids. Future work will involve an investigation of this novel class of compounds as potential biological targets and their potential as a bioisosteric relative of biologically active hydroxamic acids.

## Experimental Section

^1^H and ^13^C{^1^H} NMR spectra were recorded on a Bruker 400 spectrometer. Chemical shifts (δ) are reported in parts per million (ppm) relative to residual solvent signals (^1^H NMR, 7.26 ppm for CDCl_3_, 2.50 ppm for DMSO-*d*_6_, and 3.31 ppm for CD_3_OD; ^13^C{^1^H} NMR, 77.16 ppm for CDCl_3_, 39.53 ppm for DMSO-*d*_6_, and 49.03 for CD_3_OD). ^13^C{^1^H} NMR spectra were acquired with the ^1^H broad band decoupled mode. Coupling constants (*J*) are in hertz (Hz). Melting points were measured using a Stuart scientific melting point apparatus and were uncorrected. Infrared spectra (IR) were recorded with KBr discs using a Bruker Tensor27 FT-IR instrument. High-resolution mass spectra were obtained on a Waters Micromass GCT PremierMS spectrometer or on a Bruker microTOF-Q III LC-MS spectrometer (APCI method). Optical rotations were measured on a PerkinElmer 343 polarimeter. HPLC chromatograms was recorded on a YMC-Triart Phenyl 150 × 4.6 mm column using a 5 μL injection volume (60:40 MeCN/H_2_O) at two different wavelengths of 190 and 254 nm, respectively. The purity of the final products was verified by HPLC analysis and ^1^H and ^13^C{^1^H} NMR spectroscopy. Analytical-grade solvents and commercially available reagents were used as received. Dry DCM was purchased from Sigma-Aldrich. Reactions were monitored by TLC (Merck, silica gel 60 F254). Flash column chromatography was performed using silica gel 60 (0.040–0.063 mm, 230–400 mesh). Substituted arylazides **4a**–**j**^28^ and pyridyl azides **2a**–**c** were prepared according to reported procedures.^29,30^ β-Ketoesters **1a**–**c** and modified ketoesters **7a**–**c** were purchased from Sigma-Aldrich and used without further purification. 5-Hydroxy-1,2,3-triazoles **5a**, **5e**, **5f**, and **5g** were synthesized via phase-transfer catalysis according to GP3 and via DBU-promoted synthesis according to GP2, while 5-hydroxy-1,2,3-triazoles **5b**, **5c**, **5d**, and **5k** were synthesized according to GP2 via a DBU-promoted synthesis. 5-Methyl-1,2,3-triazoles **3a**–**b** were synthesized according to the GP1 procedure. 5-Methyl-1,2,3-triazole **3c** was synthesized according to a modified version of GP1. Long-chained 5-hydroxy-1,2,3-triazoles based SAHA analogs **10b**–**c** were synthesized according to GP6 via long-chained 5-hydroxy-1,2,3-triazole precursors **8a**–**c**, which were synthesized according to GP4. The long-chained 5-hydroxy-1,2,3-triazole-based SAHA analog **10a** was synthesized according to a modified version of GP4.

### General Procedure for the DBU-Promoted Synthesis of 5-Methyl-1,2,3-triazoles **3a** and **3b** (GP1)

To a solution of pyridyl azides **2a** or **2b** (0.5 mmol, 1 equiv) and β-ketoester **1a** (0.6 mmol, 1.2 equiv) in MeCN (2.5 mL, 0.2M) was added DBU (0.6 mmol, 1.2 equiv), and the reaction mixture was stirred at 50 °C in an oil bath overnight. The crude mixture was evaporated under vacuum and purified via flash column chromatography (MeOH/DCM/AcOH 90:10:0.1) to afford title compounds **3a** and **3b** as solids.

#### Ethyl 5-Methyl-1-(pyridin-4-yl)-1*H*-1,2,3-triazole-4-carboxylate **3a**

Yellow solid (93 mg, 80% yield). ^1^H NMR (400 MHz, CDCl_3_): δ 8.88 (d, *J* = 6.1 Hz, 2H), 7.51 (d, *J* = 6.1 Hz, 2H), 4.48 (q, *J* = 7.1 Hz, 2H), 2.72 (s, 3H), 1.46 (t, *J* = 7.1 Hz, 3H). ^13^C{^1^H} NMR (101 MHz, CDCl_3_): δ 161.4, 151.7, 142.5, 138.7, 118.7, 61.4, 14.4, 10.3. IR (KBr, cm^–1^): 3278, 3132, 3100, 1748, 1560, 1480. mp 120–120.7 °C. HRMS (ESI) *m*/*z*: [M + H]^+^ calcd for C_11_H_12_N_4_O_2_ 232.0960, found 232.0948.

#### Ethyl 5-Methyl-1-(pyridin-3-yl)-1*H*-1,2,3-triazole-4-carboxylate **3b**

Yellow solid (84 mg, 72% yield). ^1^H NMR (400 MHz, CDCl_3_): δ 8.83 (d, *J* = 4.8 Hz, 1H), 8.78 (s, 1H), 7.89 (d, *J* = 8.1 Hz, 1H), 7.58 (dd, *J* = 8.1, 4.8 Hz, 1H), 4.48 (q, *J* = 7.1 Hz, 2H), 2.65 (s, 3H), 1.46 (t, *J* = 7.1 Hz, 3H). ^13^C{^1^H} NMR (101 MHz, CDCl_3_): δ 161.5, 151.2, 145.9, 139.1, 137.2, 132.9, 132.4, 124.3, 77.1, 77.1, 76.8, 61.3, 14.4, 9.9. All analytical data are consistent with those reported in the literature.^31^

### Synthesis of Ethyl 5-Methyl-1-(pyridin-2-yl)-1*H*-1,2,3-triazole-4-carboxylate **3c**

To a solution of **2c** (100 mg, 0.83 mmol, 1 equiv) in DMSO (1.4 mL, 0.6M) were added **1a** (141 ul, 1 mmol, 1.2 equiv), DBU (150 ul, 1 mmol, 1.2 equiv), and Cu(OTf)_2_·C_6_H_5_CH_3_ (43 mg, 0.083 mmol, 0.1 equiv). The reaction mixture turned from light brown to black upon the addition of the catalyst and was stirred for 8 h at reflux in an oil bath. After 8 h, TLC showed the complete consumption of **2c**, and the mix was cooled to room temperature and extracted with DCM/H_2_O three times. The collected organic phases were filtered through a Celite pad and concentrated in vacuo. The crude product was purified by flash column chromatography (DCM/AcOEt 90:10) to afford the product **3c** in a modest yield (58 mg, 50% yield) as a yellow oil. TLC showed the product to be visible as a brilliant purple spot under an UV lamp at a short wavelength. All analytical data are consistent with those reported in the literature. ^1^H NMR (400 MHz, CDCl_3_): δ 8.58 (d, *J* = 4.6 Hz, 1H), 7.95 (m, 2H), 7.42 (m, 1H), 4.46 (q, *J* = 7.1 Hz, 2H), 2.91 (s, 3H), 1.44 (t, *J* = 7.1 Hz, 3H). ^13^C{^1^H} NMR (101 MHz, CDCl_3_): δ 160.7, 149.2, 147.5, 138.7, 138.1, 136.3, 123.1, 117.34, 60.1, 13.4, 10.0.

### General Procedure for the DBU-Promoted Synthesis of 5-Hydroxy-1,2,3-triazoles **5a**–**k** (GP2)

To a solution of aryl azides **4** (0.5 mmol, 1 equiv) and β-ketoester **1** (0.6 mmol, 1.2 equiv) in MeCN (0.2M) was added DBU (0.6 mmol, 1.2 equiv), and the reaction mixture was stirred at 50 °C in an oil bath overnight. The crude mixture was evaporated under vacuum and purified via flash column chromatography (MeOH/DCM/AcOH 90:10:0.1) to afford title compounds **5a**–**k** as solids. In some cases after chromatography, products **5a**–**k** still contained traces of the DBU salt, which were easily removed by the trituration of the solid with a minimum quantity of water.

### General Procedure for the Synthesis of 5-Hydroxy-1,2,3-triazoles **5a** and **5e**–**g** via Phase-Transfer Catalysis (GP3)

To a solution of aryl azides **4a**, **4e**–**g** (1.2 mmol, 1 equiv), and β-ketoester **1** (1.2 mmol,1 equiv) in diethyl ether (0.2M) were added tetrabutylammonium bromide (0.11 mmol, 10 mol %) and finely ground KOH (2.4 mmol, 2 equiv) at room temperature. After 4 h of vigorous stirring, the white precipitate was collected by vacuum filtration. The solid, a potassium salt of **5**, was dispersed in 1 mL of MeCN, and acetic acid was added until dissolution. The mixture was evaporated and purified via flash column chromatography (MeOH/DCM 1:9) to afford the products **5a** and **5e**–**g**.

#### 5-Hydroxy-4-methyl-1-phenyl-1,2,3-triazole **5a**

Prepared according both GP2 and GP3 to provide **5a** as a white solid (70 mg, 80% yield and 201 mg, 95% yield, respectively). ^1^H NMR (400 MHz, CDCl_3_): δ 12.82 (bs, 1H), 7.80 (d, *J* = 7.7 Hz, 2H), 7.44 (t, *J* = 7.7 Hz, 2H), 7.39 (t, *J* = 7.4 Hz, 1H), 2.25 (s, 3H). ^13^C{^1^H} NMR (101 MHz, CDCl_3_): δ 152.2, 135.8, 129.1, 128.4, 122.4, 119.6, 8.3. IR (KBr, cm^–1^): 1852, 1493, 759. mp 140–140.1 °C. HRMS (ESI) *m*/*z*: [M + H]^+^ calcd for C_9_H_10_N_3_O 176.0824, found 176.0819.

#### 5-Hydroxy-4-methyl-1-(4-bromophenyl)-1,2,3-triazole **5b**

Prepared according to GP_2_. Off-white solid (110 mg, 87% yield). ^1^H NMR (400 MHz, DMSO-*d*_6_): δ 11.76 (bs, 1H), 7.70–7.74 (m, 4H), 2.18 (s, 3H). ^13^C{^1^H} NMR (101 MHz, DMSO-*d*_6_): δ 147.2, 135.5, 132.8, 124.2, 123.2, 121.0, 9.6. IR (KBr, cm^–1^): 2389, 1991, 1493. mp 154–154.4 °C. HRMS (ESI) *m*/*z*: [M + H]^+^ calcd for C_9_H_9_BrN_3_O 253.9929, found 253.9935.

#### 5-Hydroxy-4-methyl-1-(4-methoxyphenyl)-1,2,3-triazole **5c**

Prepared according to GP_2_. White solid (64 mg, 62% yield). ^1^H NMR (400 MHz, DMSO-*d*_6_): δ 9.67 (bs, 1H), 7.57 (d, *J* = 9.0 Hz, 1H), 7.09 (d, *J* = 9.0 Hz, 1H), 3.82 (s, 1H), 2.17 (s, 1H). ^13^C{^1^H}NMR (101 MHz, DMSO-*d*_6_): δ 159.3, 146.8, 129.2, 124.5, 123.2, 114.9, 55.9, 9.7. IR (KBr, cm^–1^): 2616, 1522, 1257. mp 153–154 °C. HRMS (ESI) *m*/*z*: [M + H]^+^ calcd for C_10_H_12_N_3_O_2_ 206.0930, found 206.0928.

#### 5-Hydroxy-4-methyl-1-(2-phenylphenyl)-1,2,3-triazole **5d**

Prepared according to GP_2_. Off-white solid (95 mg, 75% yield). ^1^H NMR (400 MHz, DMSO-*d*_6_): δ 9.87 (bs, 1H), 7.70–7.62 (m, 1H), 7.61–7.52 (m, 2H), 7.49–7.41 (m, 1H), 7.36–7.25 (m, 3H), 7.18–7.05 (m, 2H), 2.05 (s, 3H). ^13^C{^1^H} NMR (101 MHz, DMSO-*d*_6_): δ 158.9, 147.9, 139.2, 138.3, 133.1, 131.2, 130.7, 128.9, 128.8, 128.5, 127.9, 122.0, 9.6. IR (KBr, cm^–1^): 2315, 1597, 1478, 1271, 1232. mp 143–143.7 °C. HRMS (ESI) *m*/*z*: [M + H]^+^ calcd for C_15_H_14_N_3_O 252.1137, found 252.1129.

#### 5-Hydroxy-4-methyl-1-(1-naphtyl)-1,2,3-triazole **5e**

Prepared according to GP_2_. White solid (89 mg, 79% yield). ^1^H NMR (400 MHz, DMSO-*d*_6_): δ 8.16 (d, *J* = 8.2 Hz, 1H), 8.10 (d, *J* = 7.8 Hz, 1H), 7.73–7.56 (m, 4H), 7.36 (d, *J* = 8.2 Hz, 1H), 2.28 (s, 3H). ^13^C{^1^H} NMR (101 MHz, DMSO-*d*_6_): δ 148.3, 134.1, 131.9, 130.4, 129.4, 128.6, 128.0, 127.3, 125.9, 125.6, 123.1, 122.4, 9.8. IR (KBr, cm^–1^): 3236, 2980, 2867, 2167. mp 119–119.3 °C. HRMS (ESI) *m*/*z*: [M + H]^+^ calcd for C_13_H_12_N_3_O 226.0980, found 226.0972.

#### 5-Hydroxy-4-methyl-1-(4-nitrophenyl)-1,2,3-triazole **5f**

Prepared according both GP_2_ and GP_3_. Yellow solid (59 mg, 53% yield and 202 mg, 76% yield, respectively). ^1^H NMR (400 MHz, DMSO-*d*_6_): δ 12.45 (bs, 1H), 8.42 (d, *J* = 9.1 Hz, 2H), 8.13(d, *J* = 9.1 Hz, 2H), 2.19 (s, 3H). ^13^C{^1^H} NMR (101 MHz, DMSO-*d*_6_): δ 146.3, 141.3, 125.6, 125.2, 121.9, 120.6, 9.51. IR (KBr, cm^–1^): 3350, 2350, 1517, 1330. mp 156–157.2 °C. HRMS (ESI) *m*/*z*: [M + H]^+^ calcd for C_9_H_9_N_4_O_3_ 221.0675, found 221.0679.

#### 5-Hydroxy-4-methyl-1-(4-methyl-2-nitrophenyl)-1,2,3-triazole **5g**

Prepared according both GP_2_ and GP_3_. Yellow solid (66 mg, 56% yield and 234 mg, 83% yield, respectively). ^1^H NMR (400 MHz, CD_3_OD): δ 8.00 (s, 1H), 7.71 (d, *J* = 8.0 Hz, 1H), 7.57 (d, *J* = 8.0 Hz, 1H), 2.54 (s, 3H), 2.22 (s, 3H). ^13^C{^1^H} NMR (101 MHz, CD_3_OD): δ 145.8, 143.4, 137.0, 135.7, 129.6, 126.7, 126.6, 123.9, 21.0, 8.4. IR (KBr, cm^–1^): 3088, 2921, 1183, 759. mp 112–112.9 °C. HRMS (ESI) *m*/*z*: [M + H]^+^ calcd for C_10_H_11_N_4_O_3_ 235.0831, found 235.0822.

#### 5-Hydroxy-4-benzyl-1-phenyl-1,2,3-triazole **5k**

Prepared according to GP_2_. White solid (95 mg, 75% yield). ^1^H NMR (400 MHz, DMSO-*d*_6_): δ 12.30 (bs, 1H), 7.75 (d, *J* = 7.5 Hz, 2H), 7.57–7.50 (m, 2H), 7.46–7.40 (m, 1H), 7.29–7.24 (m, 4H), 7.22–7.15 (m, 1H), 3.96 (s, 2H). ^13^C{^1^H} NMR (101 MHz, DMSO-*d*_6_): δ 147.5, 140.2, 136.0, 129.3, 128.3, 128.2, 127.7, 126.2, 125.9, 122.0, 29.3. IR (KBr, cm^–1^): 2783, 1596, 1493. mp 146–146.5 °C. HRMS (ESI) *m*/*z*: [M + H]^+^ calcd for C_15_H_14_N_3_O 252.1137, found 252.1130.

### General Procedure for the Synthesis of Long-Chained 5-Hydroxy-1,2,3-triazole Esters **8a**–**c** (GP4)

To a mixture of commercially available cyclic ketoesters **7a**–**c** (2.4 mmol, 1.2 equiv) and azidobenzene **4a** (2.0 mmol, 1 equiv) in MeCN (0.2M) was added DBU (2.4 mmol, 1.2 equiv), and the reaction mixture was stirred at 60 °C in an oil bath overnight. The crude mixture was purified by silica flash chromatography (AcOEt/petroleum ether gradient from 1:2 to 1:0) to afford 1,2,3-triazole esters **8a**–**c** as solids. Better yields were obtained when using the same protocol in solvent-free conditions.

#### Ethyl 5-(5-Hydroxy-1-phenyl-1,2,3-triazol-4-yl)pentanoic Ester **8a**

Off-white solid (153 mg, 44% yield in solvent-free conditions versus 101 mg, 29% yield with solvent present). ^1^H NMR (400 MHz, CDCl_3_): δ 13.50 (bs, 1H), 7.85 (d, *J* = 7.7 Hz, 2H), 7.46 (t, *J* = 6.9 Hz, 2H), 7.36 (t, *J* = 7.4 Hz, 1H), 4.06 (q, *J* = 7.2 Hz, 2H), 2.70 (t, *J* = 7.1 Hz, 2H), 2.24 (t, *J* = 7.2 Hz, 2H), 1.75–1.53 (m, 4H), 1.19 (t, *J* = 7.1 Hz, 3H). ^13^C{^1^H} NMR (101 MHz, CDCl_3_): δ 173.9, 152.0, 135.7, 129.1, 128.4, 123.3, 122.4, 60.4, 33.8, 27.8, 24.1, 22.4, 14.2. IR (KBr, cm^–1^): 2507, 1734, 1604, 1570. mp 98–99.1 °C. HRMS (ESI) *m*/*z*: [M + H]^+^ calcd for C_15_H_20_N_3_O_3_290.1505, found 290.1502.

#### Methyl 6-(5-Hydroxy-1-phenyl-1,2,3-triazol-4-yl)hexanoic Ester **8b**

Off-white solid (275 mg, 79% yield in solvent-free conditions). ^1^H NMR (400 MHz, CDCl_3_): δ 10.61 (bs, 1H), 7.87 (d, *J* = 7.7 Hz, 2H), 7.49–7.40 (m, 2H), 7.38–7.33 (m, 1H), 3.61 (s, 3H), 2.65 (t, *J* = 7.6 Hz, 2H), 2.22 (t, *J* = 7.4 Hz, 2H), 1.69–1.61 (m, 2H), 1.60–1.51 (m, 2H), 1.34–1.24 (m, 2H). ^13^C{^1^H}NMR (101 MHz, CDCl_3_): δ 174.5, 151.5, 136.0, 129.0, 128.2, 124.3, 122.2, 51.6, 33.8, 28.5, 28.2, 24.3, 22.7. IR (KBr, cm^–1^): 2857, 2512, 1744, 1606. mp 112–112.6 °C. HRMS (ESI) *m*/*z*: [M + H]^+^ calcd for C_15_H_20_N_3_O_3_ 290.1505, found 290.1492.

#### Ethyl 7-(5-Hydroxy-1-phenyl-1,2,3-triazol-4-yl)heptanoic ester **8c**

Off-white solid (145 mg, 38% yield in solvent-free conditions). ^1^H NMR (400 MHz, CDCl_3_): δ 13.43 (bs, 1H), 7.90 (d, *J* = 7.6 Hz, 2H), 7.50–7.42 (m, 2H), 7.40–7.32 (m, 1H), 4.08 (q, *J* = 7.1 Hz, 2H), 2.64 (t, *J* = 7.6 Hz, 2H), 2.18 (t, *J* = 7.5 Hz, 2H), 1.71–1.58 (m, 2H), 1.55–1.45 (m, 2H), 1.33–1.10 (m, 7H). ^13^C{^1^H} NMR (101 MHz, CDCl_3_): δ 174.0, 151.9, 136.1, 129.0, 128.1, 124.1, 122.2, 60.3, 34.2, 28.8, 28.7, 28.5, 24.8, 23.0, 14.2. IR (KBr, cm^–1^): 2869, 2524, 1732, 1599. mp 100–101.3 °C. HRMS (ESI) *m*/*z*: [M + H]^+^ calcd for C_17_H_24_N_3_O_3_ 318.1818, found 318.1816.

### General Procedure for the Synthesis of Long-Chained 5-Hydroxy-1,2,3-triazolecarboxylic acids **11a**–**c** (GP5)

To a dispersion of 1,2,3-triazole esters **8a**–**c** (0.5 mmol, 1 equiv) in water (0.1M) at 0 °C was added KOH pellets (5.0 mmol, 10 equiv). Upon the complete dissolution of KOH and the ester, the ice bath was removed, and the reaction mixture was vigorously stirred for 2 h at room temperature. The reaction mixture was again cooled to 0 °C, and HCl (37% aq.) was added dropwise to reach pH 1 and precipitate out the free form of the desired compound. The latter was collected by vacuum filtration and dried under vacuum to afford 5-hydroxy-1,2,3-triazolecarboxylic acids **11a**–**c** in their pure form.

#### 5-(5-Hydroxy-1-phenyl-1,2,3-triazol-4-yl)pentanoic Acid **11a**

White solid (79 mg, 60% yield). ^1^H NMR (400 MHz, DMSO-*d*_6_): δ 11.95 (bs, 1H), 11.38 (bs, 1H), 7.65 (d, *J* = 7.3 Hz, 2H), 7.56–7.45 (m, 2H), 7.44–7.34 (m, 1H), 2.51 (t, *J* = 6.8 Hz, 2H), 2.19 (t, *J* = 6.8 Hz, 2H), 1.65–1.42 (m, 4H). ^13^C{^1^H}NMR (101 MHz, DMSO-*d*_6_): δ 174.5, 135.8, 129.4, 128.0, 122.2, 33.5, 28.4, 24.2, 23.1. IR (KBr, cm^–1^): 3340, 2350, 1700, 1601, 1562. mp 122–122.8 °C. HRMS (ESI) *m*/*z*: [M + H]^+^ calcd for C_13_H_16_N_3_O_3_ 262.1192, found 262.1184.

#### 6-(5-Hydroxy-1-phenyl-1,2,3-triazol-4-yl)hexanoic Acid **11b**

White solid (122 mg, 88% yield). ^1^H NMR (400 MHz, DMSO-*d*_6_): δ 11.80 (s, 2H), 7.67 (d, *J* = 7.8 Hz, 2H), 7.53–7.46 (m, 2H), 7.44–7.36 (m, 1H), 2.51 (t, *J* = 7.6 Hz, 2H), 2.17 (t, *J* = 7.4 Hz, 2H), 1.61–1.44 (m, 4H), 1.35–1.23 (m, 2H). ^13^C{^1^H} NMR (101 MHz, DMSO-*d*_6_): δ 174.6, 135.9, 129.3, 127.9, 122.2, 33.7, 28.6, 28.3, 24.4, 23.2. IR (KBr, cm^–1^): 3395, 2938, 1724, 1629, 1599. mp 115–115.3 °C. HRMS (ESI) *m*/*z*: [M + H]^+^ calcd for C_14_H_18_N_3_O_3_ 276.1348, found 276.1333.

#### 7-(5-Hydroxy-1-phenyl-1,2,3-triazol-4-yl)heptanoic Acid **11c**

White solid (118 mg, 81% yield). ^1^H NMR (400 MHz, DMSO-*d*_6_): δ 11.89 (bs, 2H), 7.71 (d, *J* = 7.7 Hz, 2H), 7.58–7.50 (m, 2H), 7.48–7.39 (m, 1H), 2.58–2.52 (m, 2H), 2.20 (t, *J* = 7.3 Hz, 2H), 1.63–1.54 (m, 2H), 1.54–1.44 (m, 2H), 1.38–1.27 (m, 4H). ^13^C{^1^H} NMR (101 MHz, DMSO-*d*_6_): δ 174.6, 135.8, 129.3, 128.0, 122.2, 33.7, 28.7, 28.5, 28.4, 24.5, 23.3. IR (KBr, cm^–1^): 3223, 2948, 1717, 1599. mp 117–117.4 °C. HRMS (ESI) *m*/*z*: [M + H]^+^ calcd for C_15_H_20_N_3_O_3_ 290.1505, found 290.1518.

### Synthesis of *N*-Phenyl-5-(5-hydroxy-1-phenyl-1,2,3-triazol-4-yl)pentanamide **10a**

Carboxylic acid **11a** (0.49 mmol, 1 equiv) was dissolved in thionyl chloride (0.49 M). The reaction mixture was refluxed at 60 °C for 2 h in an oil bath. The reaction mixture was evaporated under vacuum to remove the excess thionyl chloride. The crude acyl chloride was dissolved in dry DCM (1 mL), and to the solution were added aniline (0.54 mmol, 1.1 equiv) and triethylamine (0.54 mmol, 1.1 equiv). The reaction mixture was stirred at room temperature under nitrogen overnight. The crude was diluted in AcOEt (20 mL) and washed with saturated sodium bicarbonate (5 mL) and HCl (5 mL of 1 M aq.). The organic phase was dried over sodium sulfate and evaporated under vacuum. The crude amide was purified by flash column chromatography (AcOEt/petroleum ether 1:1 to 1:0) to yield the target compound **10a** in a 61% yield (101 mg) as a white solid. ^1^H NMR (400 MHz, DMSO-*d*_6_): δ 9.80 (s, 1H), 7.72 (d, *J* = 7.8 Hz, 2H), 7.64–7.51 (m, 4H), 7.48–7.39 (m, 1H), 7.32–7.23 (m, 2H), 7.06–6.97 (m, 1H), 2.67–2.58 (m, 2H), 2.42–2.31 (m, 2H), 1.75–1.60 (m, 4H). ^13^C{^1^H} NMR (101 MHz, DMSO-*d*_6_): δ 171.3, 139.4, 135.8, 129.3, 128.7, 128.0, 123.0, 122.2, 119.1, 36.4, 28.6, 24.9, 23.2. IR (KBr, cm^–1^): 2372, 1649, 1590. mp 152–153 °C. HRMS (ESI) *m*/*z*: [M + H]^+^ calcd for C_19_H_21_N_4_O_2_ 337.1665, found 337.1676.

### General Procedure for the Synthesis of Long-Chained *N*-Phenyl-5-hydroxy-1,2,3-triazoleamides **10b** and **10c** (GP6)

To a solution of carboxylic acid **11b** or **11c** (0.40 mmol, 1 equiv) in dry DCM (0.2M) in a round-bottom flask was added triethylamine (0.48 mmol, 1.2 equiv) and aniline (0.48 mmol, 1.2 equiv). The solution was added with carbonyldiimidazole (CDI) (0.48 mmol, 1.2 equiv) and stirred overnight at room temperature. The crude mixture was diluted with AcOEt (20 mL), washed with HCl (5 mL of 0.5 M aq.), dried over sodium sulfate, and evaporated under vacuum. The crude amide was purified by flash column chromatography (AcOEt/petroleum ether 1:1 to 1:0) to yield the title compounds **10b** and **10c** as white solids.

#### *N*-Phenyl-6-(5-hydroxy-1-phenyl-1,2,3-triazol-4-yl)hexanamide **10b**

White solid (49 mg, 35% yield). ^1^H NMR (400 MHz, DMSO-*d*_6_): δ 11.36 (bs, 1H), 9.86 (s, 1H), 7.71 (d, *J* = 6.7 Hz, 2H), 7.62–7.50 (m, 4H), 7.48–7.40 (m, 1H), 7.32–7.22 (m, 2H), 7.05–6.96 (m, 1H), 2.58 (t, *J* = 7.5 Hz, 2H), 2.31 (t, *J* = 7.4 Hz, 2H), 1.72–1.55 (m, 4H), 1.45–1.33 (m, 2H). ^13^C{^1^H} NMR (101 MHz, DMSO-*d*_6_): δ 171.3, 139.4, 135.9, 129.3, 128.7, 127.9, 122.9, 122.2, 119.0, 36.4, 28.7, 28.4, 25.0, 23.2. IR (KBr, cm^–1^): 2387, 1654, 1599. mp 148–149.3 °C. HRMS (ESI) *m*/*z*: [M + H]^+^ calcd for C_20_H_23_N_4_O_2_ 351.1821, found 351.1815.

#### *N*-Phenyl-7-(5-hydroxy-1-phenyl-1,2,3-triazol-4-yl)heptanamide **10c**

White solid (70 mg, 48% yield). ^1^H NMR (400 MHz, DMSO-*d*_6_): δ 9.87 (s, 1H), 7.71 (d, *J* = 7.8 Hz, 2H), 7.62–7.49 (m, 4H), 7.48–7.40 (m, 1H), 7.31–7.21 (m, 2H), 7.05–6.96 (m, 1H), 2.57 (t, *J* = 7.5 Hz, 2H), 2.30 (t, *J* = 7.4 Hz, 2H), 1.66–1.55 (m, 4H), 1.41–1.29 (m, 4H). ^13^C{^1^H} NMR (101 MHz, DMSO-*d*_6_): δ 171.3, 139.4, 135.8, 129.3, 128.7, 127.9, 122.9, 122.2, 119.0, 36.4, 28.7, 28.5 (2C), 25.1, 23.3. IR (KBr, cm^–1^): 2367, 1658, 1535. mp 142–142.6 °C. HRMS (ESI) *m*/*z*: [M + H]^+^ calcd for C_21_H_25_N_4_O_2_ 365.1978, found 365.1991

## Supporting Information Available

Copies of the ^1^H- and ^13^C{^1^H}-NMR spectra of the synthesized compounds, HPLC data of **5a**, potentiometric titration procedure for triazole **5a**, and crystallographic data of **5a** (PDF)

## Accession Codes

CCDC 2051417 contains the supplementary crystallographic data for this paper. These data can be obtained free of charge via www.ccdc.cam.ac.uk/data_request/cif, or by emailing data_request@ccdc.cam.ac.uk, or by contacting The Cambridge Crystallographic Data Centre, 12 Union Road, Cambridge CB2 1EZ, UK; fax: +44 1223 336033.
