# Supplementary material for: Preparation of Acidic 5-Hydroxy-1,2,3-triazoles via the Cycloaddition of Aryl Azides with β-Ketoesters
Source: J Org Chem. 2021 Jul 27;86(17):11354–60. doi: 10.1021/acs.joc.1c00778 (PMC8419836; doi:10.1021/acs.joc.1c00778)

Electronic Supporting Information for the paper:

Preparation of acidic 5-hydroxy-1,2,3-triazoles via cycloaddition of aryl azides with β-ketoesters.

Roberta Pacifico, Dario Destro, Malachi W. Gillick-Healy, Brian G. Kelly, Mauro F. A. Adamo*

Table of Contents

1. Potentiometric titration of 5-hydroxytriazole **5a** **S2**
2. Preparation of X-ray crystallographic analysis of compound **5a** **S3**
3. Crystal parameters and structure refinement for compound **5a** **S4**
4. HPLC chromatogram of compound **5a** **S5**
5. ^{1}H and ^{13}C{^{1}H} copies of NMR spectra **S6**

1. Potentiometric titration of 5-hydroxytriazole 5a

A 0.1 M solution of **5a** was titrated with 0.1 M NaOH, and the pH was recorded with a glass electrode. The titration curve was obtained by plotting the dataset obtained with the potentiometric titration (Figure S1). The curve showed the behaviour typical of a weak acid, with a buffer region at pH ≈ pKa. Using the graphical method we determined the equivalence point (**5a** is fully deprotonated at pH = 8.4), and consequently, the half-neutralisation point at which the pH equals the pKa value of the analyte. A pKa of 4.2 in water indicates an acid with the strength comparable to that of a carboxylic acid.

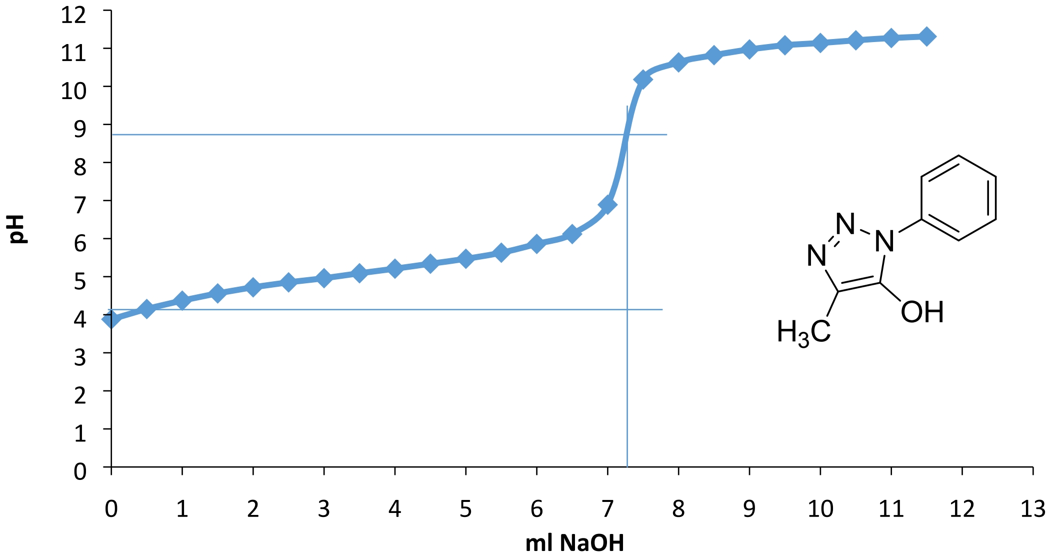

Figure S1: Potentiometric titration of 5-hydroxytriazole **5a**. Equivalence point: pH = 8.4, pKa = 4.2.

2. Preparation of X-ray crystallographic analysis of compound 5a .

A specimen of $C_9H_9N_3O$ (compound **5a,** Figure S2), approximate dimensions 0.080 mm x 0.100 mm x 0.160 mm, was used for the X-ray crystallographic analysis. The X-ray intensity data were measured (λ = 1.54178 Å) at 100(2)K on a Bruker Apex Kappa Duo with an Oxford Cobra Cryosystem low temperature device using a MiTeGen micromount. Bruker APEX software was used to correct for Lorentz and polarization effects.

A total of 1466 frames were collected. The total exposure time was 20.79 hours. The integration of the data using a monoclinic unit cell yielded a total of 5935 reflections to a maximum θ angle of 60.22° (0.89 Å resolution), of which 1240 were independent (average redundancy 4.786, completeness = 95.9%, Rint = 6.08%, Rsig = 5.10%) and 812 (65.48%) were greater than 2σ(F2). The final cell constants of a = 8.2941(6) Å, b = 6.1817(4) Å, c = 16.8539(12) Å, β = 90.258(5)°, volume = 864.12(10) Å3, are based upon the refinement of the XYZ-centroids of 1970 reflections above 20 σ(I) with 10.49° < 2θ < 118.0°. Data were corrected for absorption effects using the Multi-Scan method (SADABS). The ratio of minimum to maximum apparent transmission was 0.765. The calculated minimum and maximum transmission coefficients (based on crystal size) are 0.8880 and 0.9420.

The structure was solved with the SHELXT structure solution program using Intrinsic Phasing and refined with the SHELXL refinement package using Least Squares minimisation with Olex2, using the space group P21/n, with Z = 4 for the formula unit, C9H9N3O. The final anisotropic full-matrix least-squares refinement on F2 with 192 variables converged at R1 = 8.73%, for the observed data and wR2 = 22.58% for all data. The goodness-of-fit was 1.090. The largest peak in the final difference electron density synthesis was 0.292 e-/Å3 and the largest hole was -0.267 e-/Å3 with an RMS deviation of 0.055 e-/Å3. On the basis of the final model, the calculated density was 1.347 g/cm3 and F(000), 368 e-.

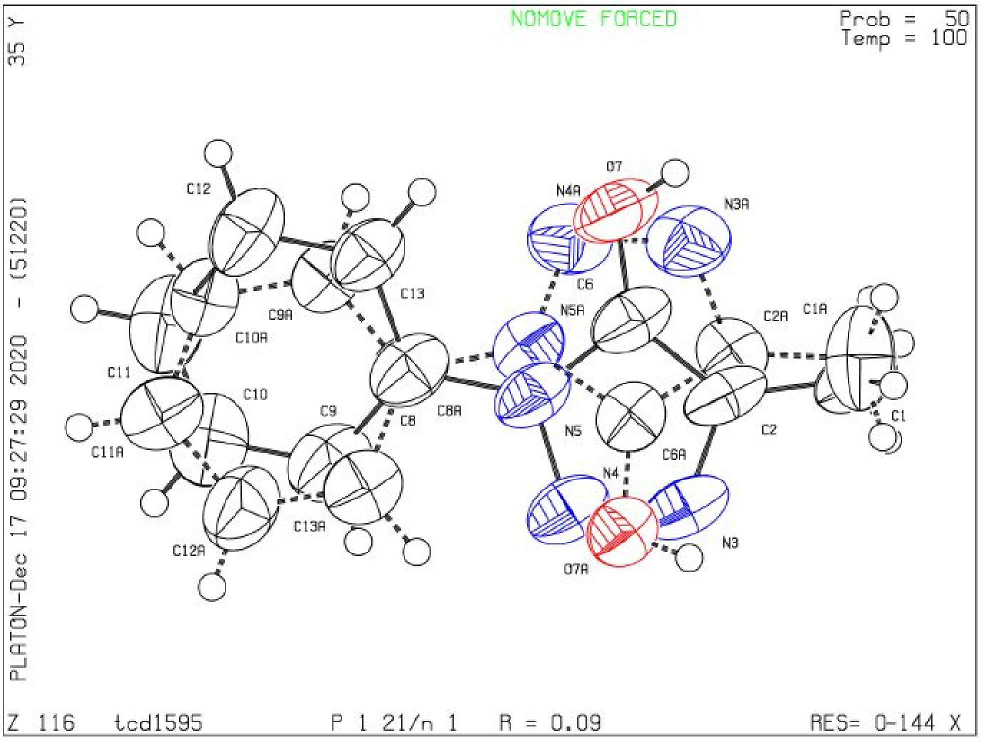

Figure S2: Thermal ellipsoid plot obtained from crystals of compound **5a**.

3. Crystal parameters and structure refinement for compound 5a

Empirical formula: $C_9H_9N_3O$

Formula weight: 175.19

Temperature: 100(2) K

Wavelength: 1.54178 Å

Crystal system: Monoclinic

Space group: P21/n

Unit cell dimensions:
- a = 8.2941(6) Å a= 90°.
- b = 6.1817(4) Å b= 90.258(5)°.
- c = 16.8539(12) Å g = 90°.

Volume: 864.12(10) Å3

Density (calculated): 1.347 mg/m^3

Absorption coefficient: 0.758 mm-1

Crystal size: 0.16 x 0.1 x 0.08 mm3

Theta range for data collection: 5.249 to 60.215°.

Index ranges: -9≤ h ≤9, -6 ≤ k ≤6, -18 ≤ l ≤16

Reflections collected: 5935

Independent reflections: 1240 [R(int) = 0.0608]

Completeness to theta: 60.215° 95.9 %

Absorption correction: Semi-empirical from equivalents

Max. and min. transmission: 0.7518 and 0.5751

Refinement method: Full-matrix least-squares on F2

Data / restraints / parameters: 1240 / 234 / 192

Goodness-of-fit on: F2 1.090

Final R indices: [I > 2σ (I)] R1 = 0.0873, wR2 = 0.1911

R indices (all data): R1 = 0.1320, wR2 = 0.2258

Largest diff. peak and hole: 0.292 and -0.267 e.Å-3

4. HPLC chromatogram of compound 5a

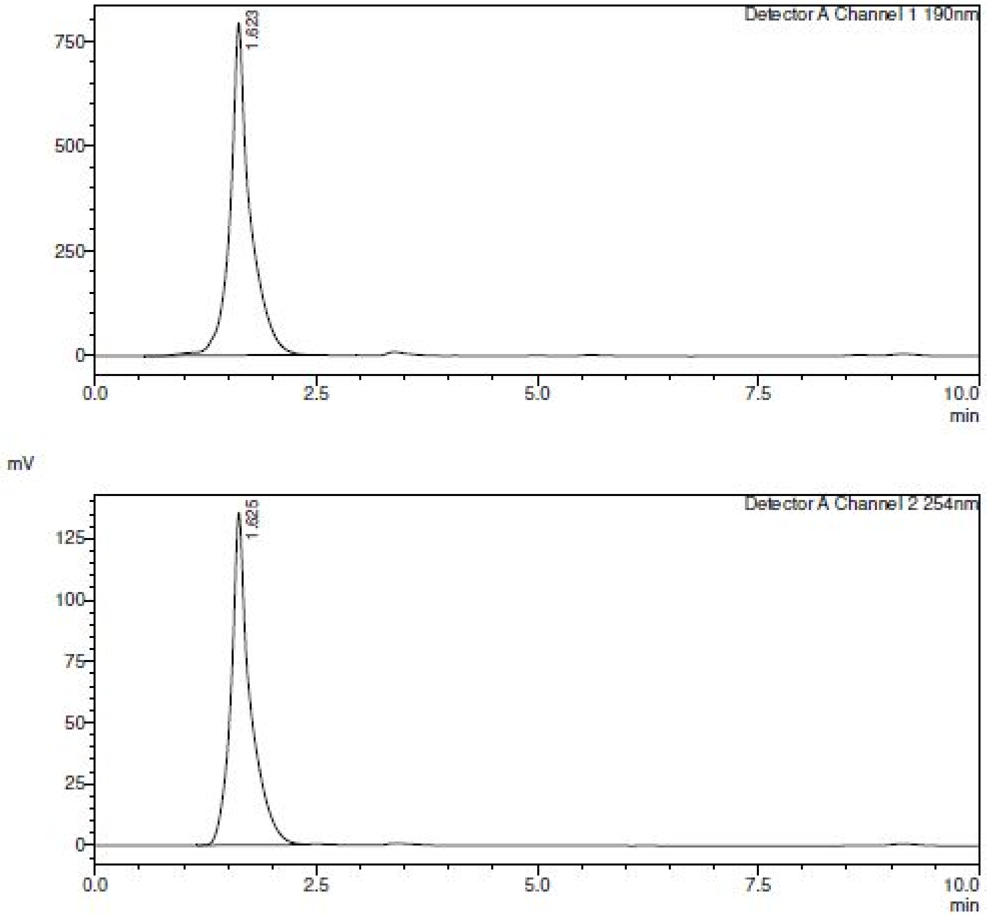

Peak#	Ret. Time	Area	Height	Conc.	Unit	Area/Height	Area%
1	1.623	12399939	790834	100.000		15.680	100.000
Total		12399939	790834				100.000

Detector A Channel 2 254nm

Peak#	Ret. Time	Area	Height	Conc.	Unit	Area/Height	Area%
1	1.625	2033623	134877	100.000		15.078	100.000
Total		2033623	134877				100.000

5. ^{1}H NMR and ^{13}C NMR Spectra

Compound **3a** (400 MHz, $CDCl_3$)

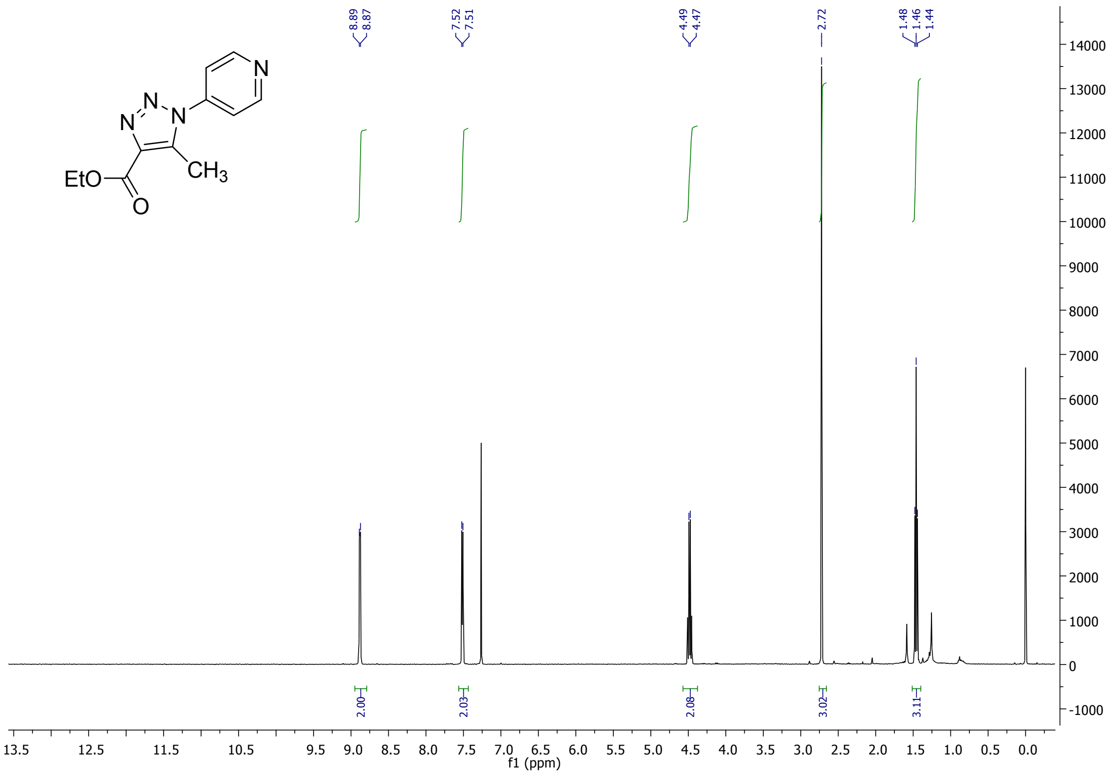

Compound **3a** $^{13}C\{^{1}H\}$ NMR (101 MHz, $CDCl_3$)

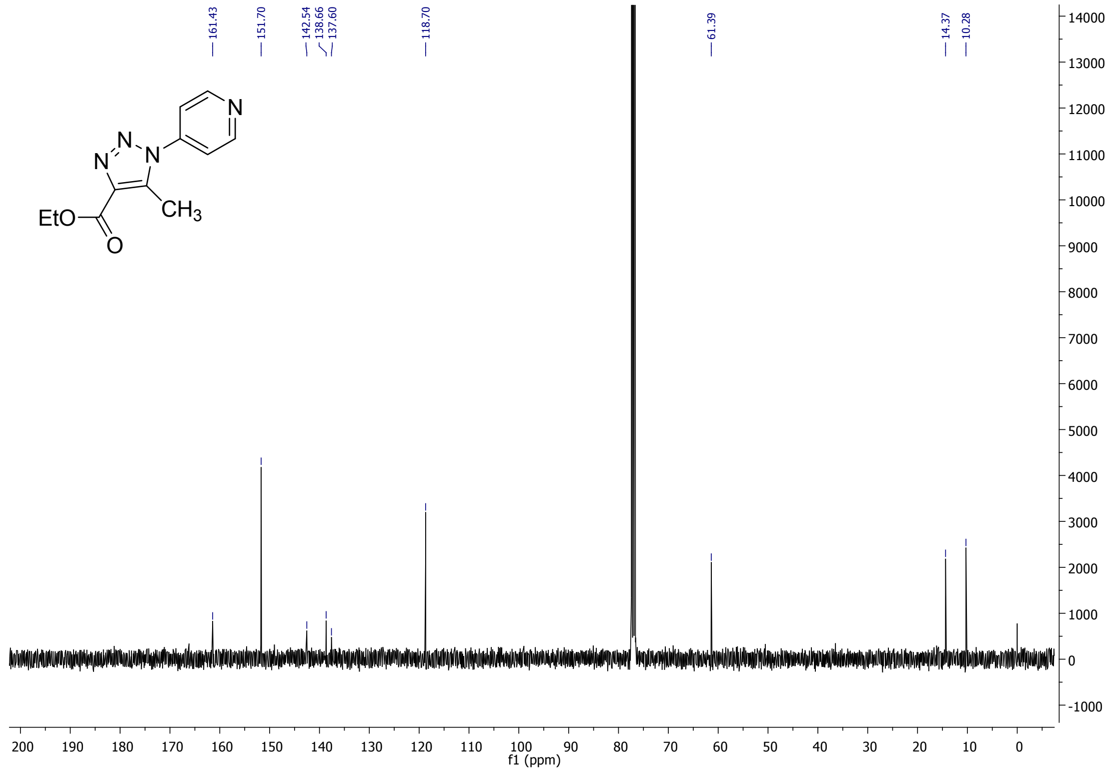

Compound **3b** (400 MHz, $CDCl_3$)

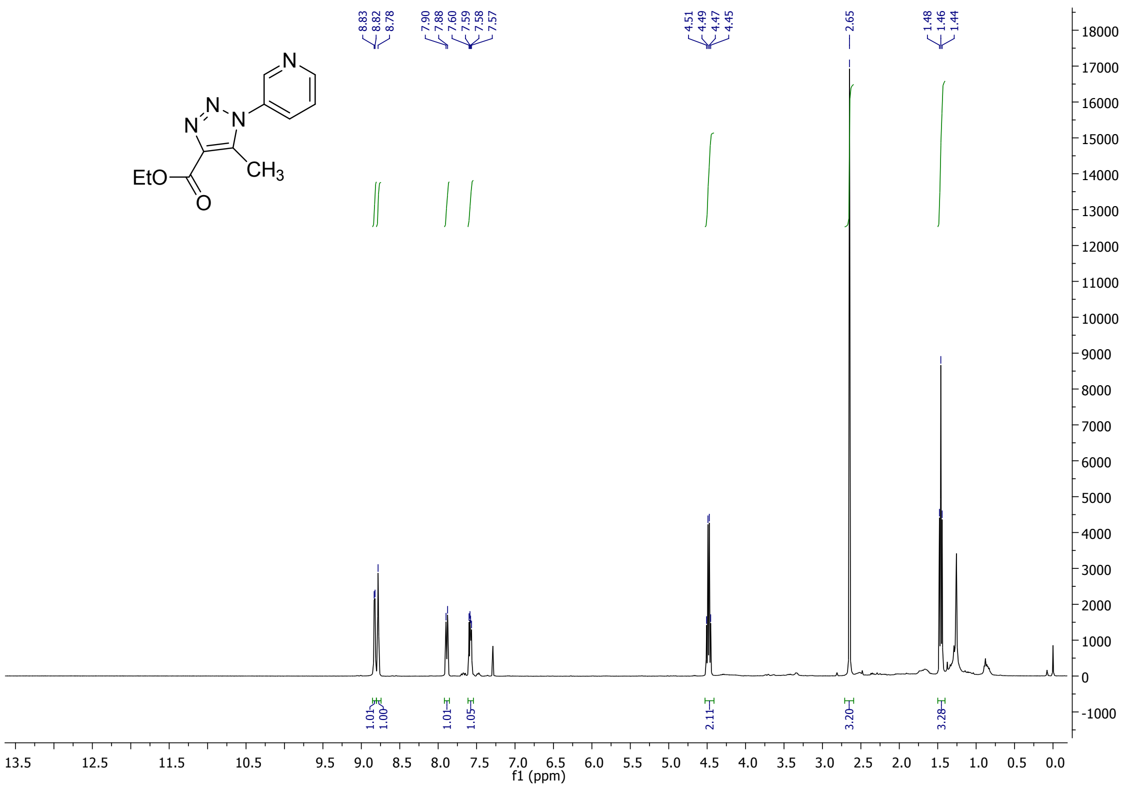

Compound **3b** $^{13}C\{^{1}H\}$ NMR (101 MHz, $CDCl_3$)

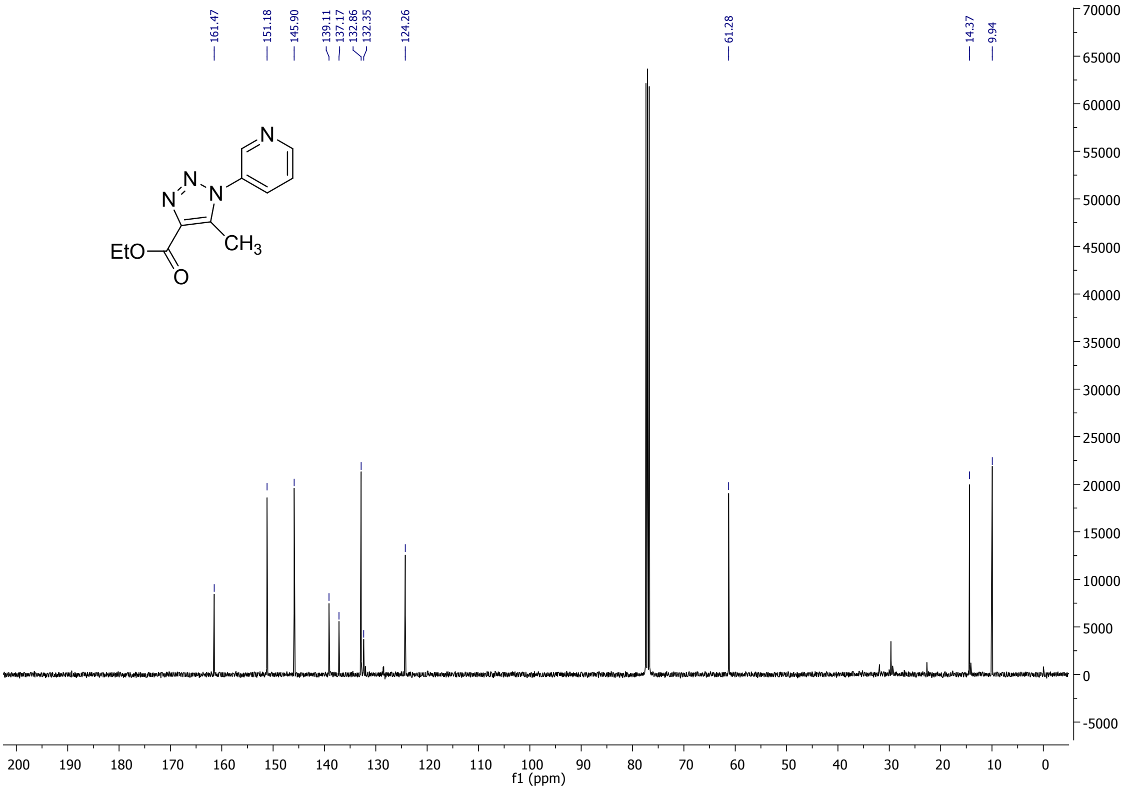

Compound **3c** (400 MHz, $CDCl_3$)

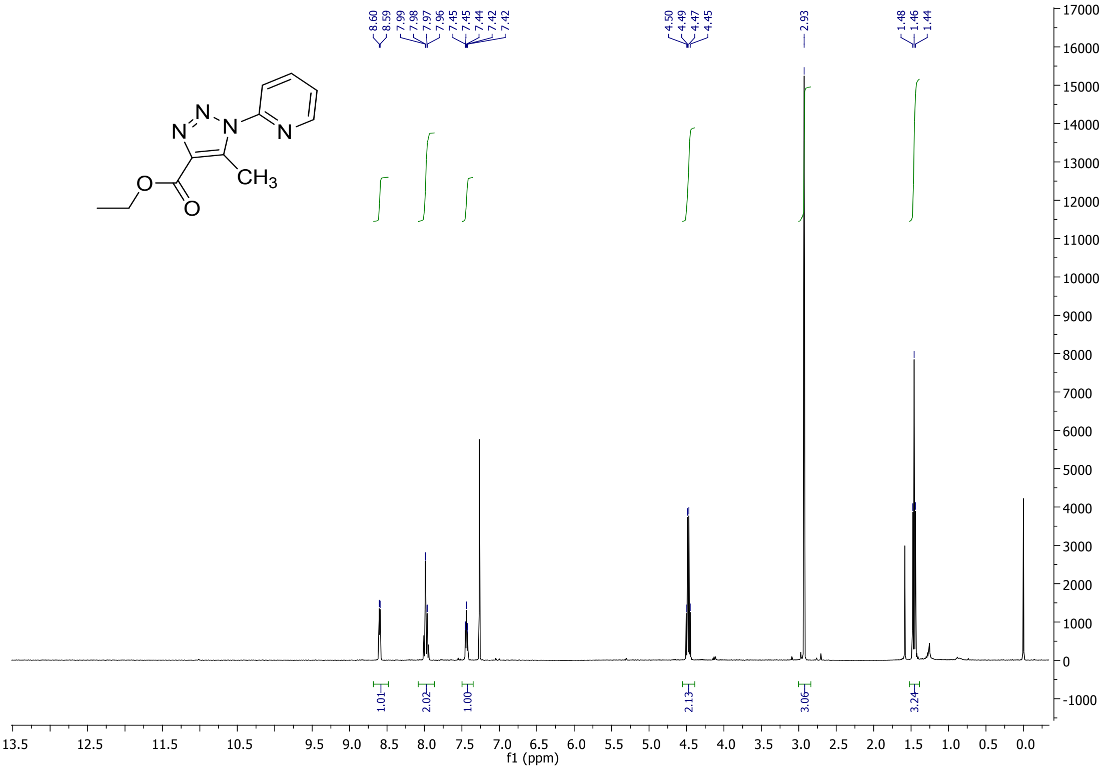

Compound **3c** $^{13}C\{^1H\}$ NMR (101 MHz, $CDCl_3$)

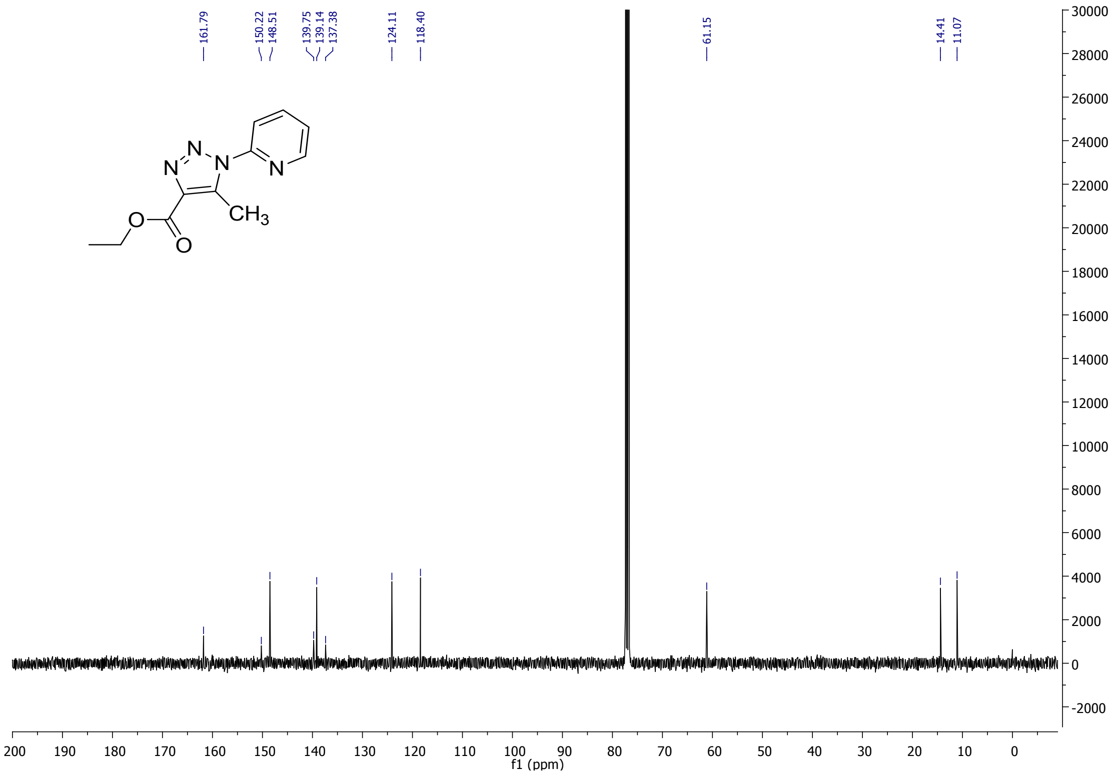

Compound **5a** (400 MHz, $CDCl_3$)

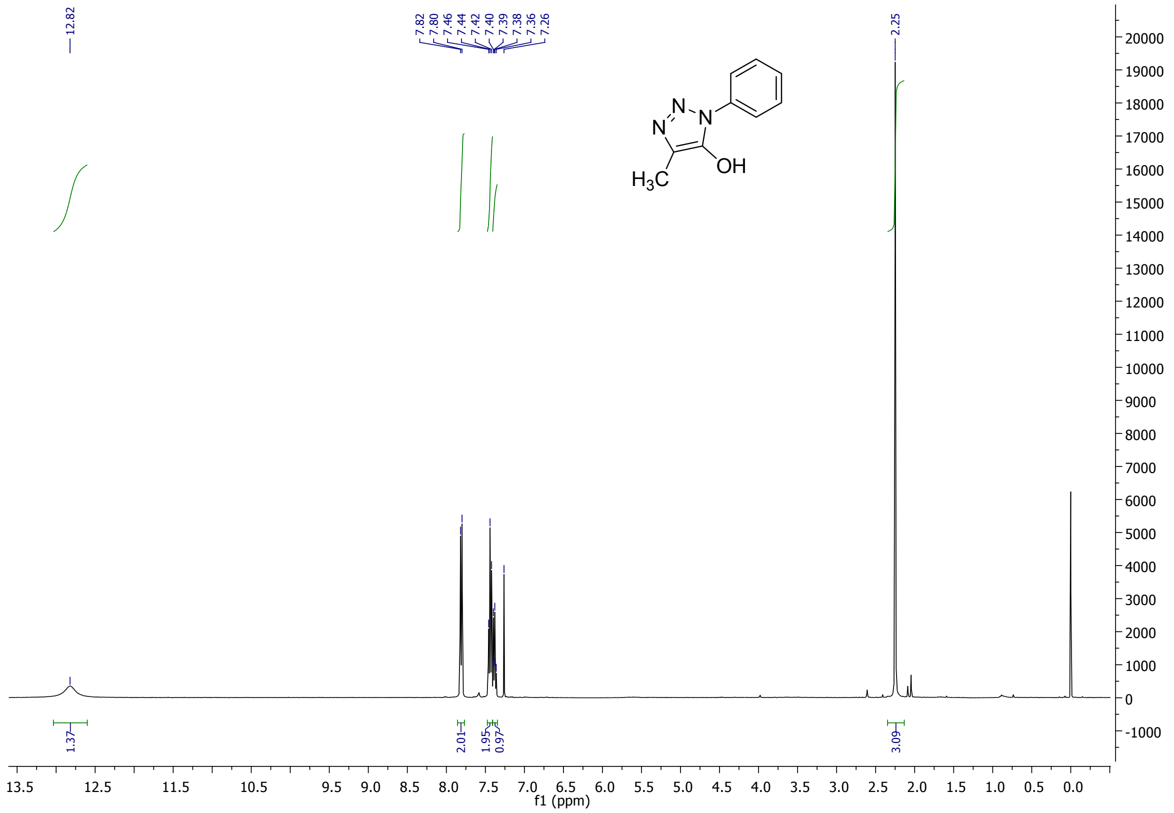

Compound **5a** $^{13}C\{^1H\}$ NMR (101 MHz, $CDCl_3$)

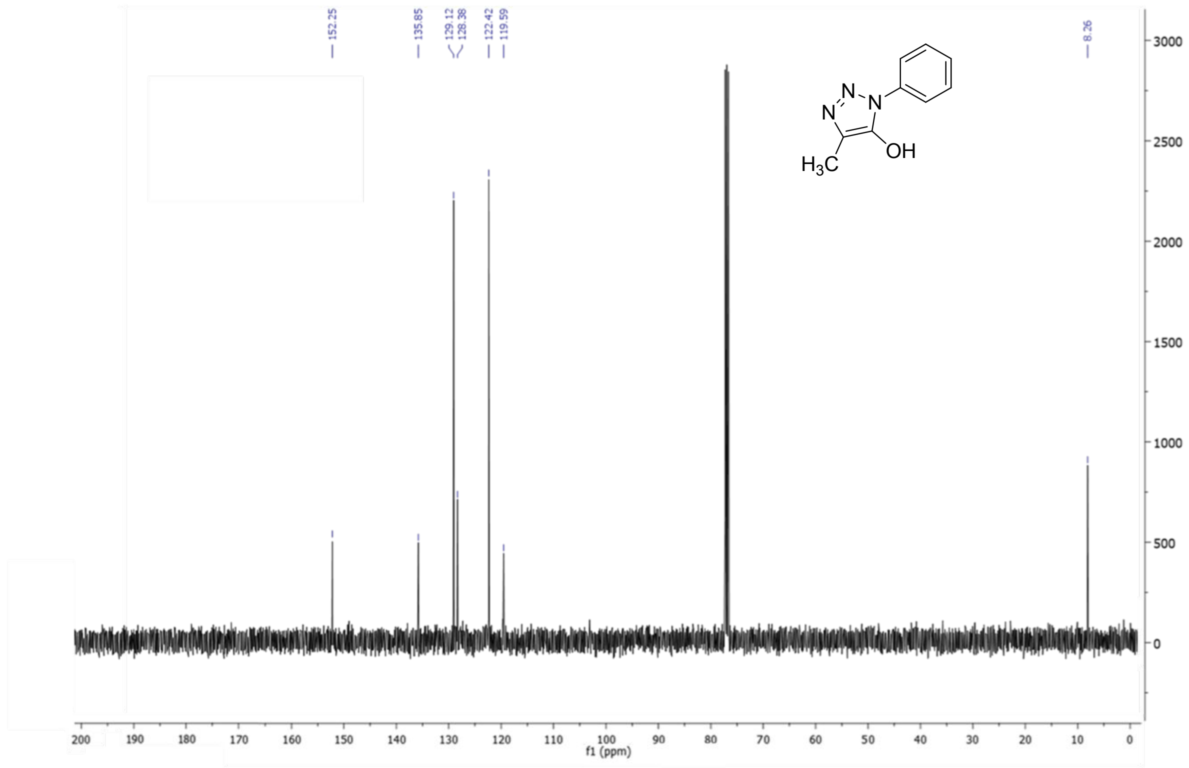

Compound **5b** (400 MHz, DMSO-d$_6$)

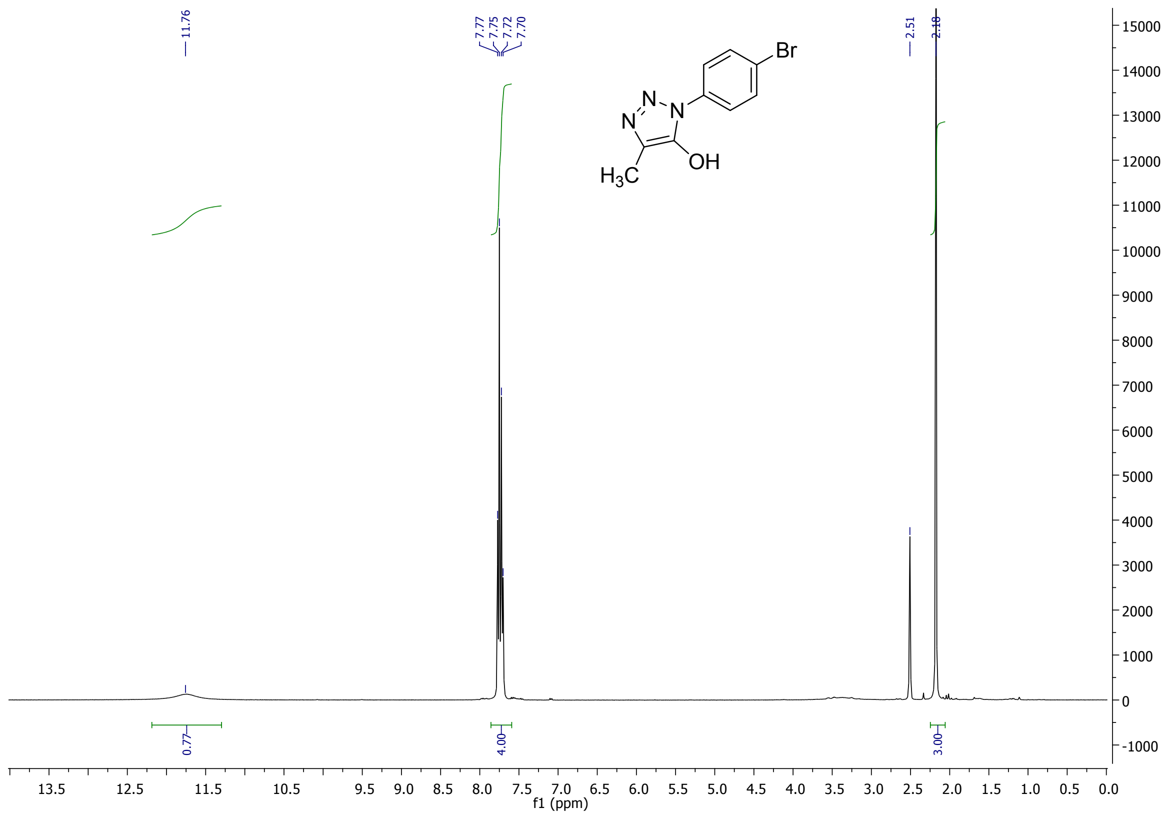

Compound **5b** $^{13}C\{^1H\}$ NMR (101 MHz, DMSO-d$_6$)

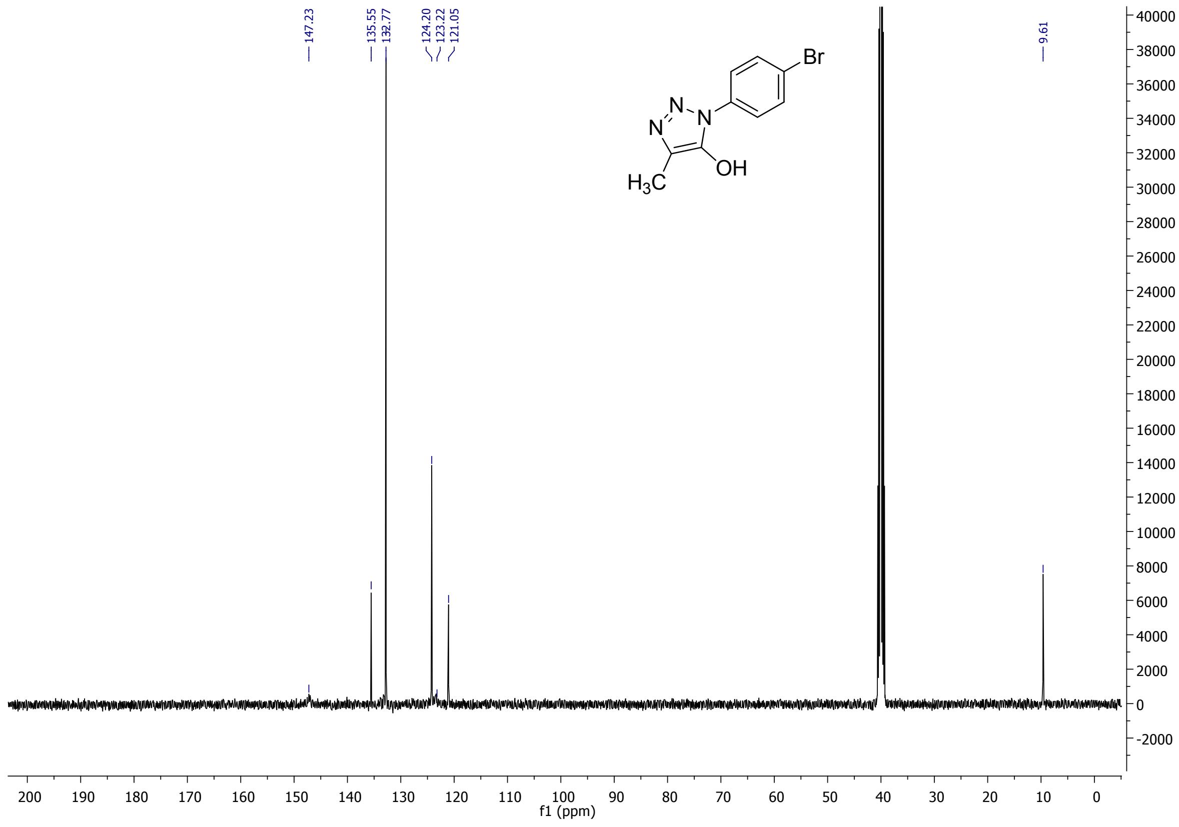

Compound **5c** (400 MHz, DMSO-d$_6$)

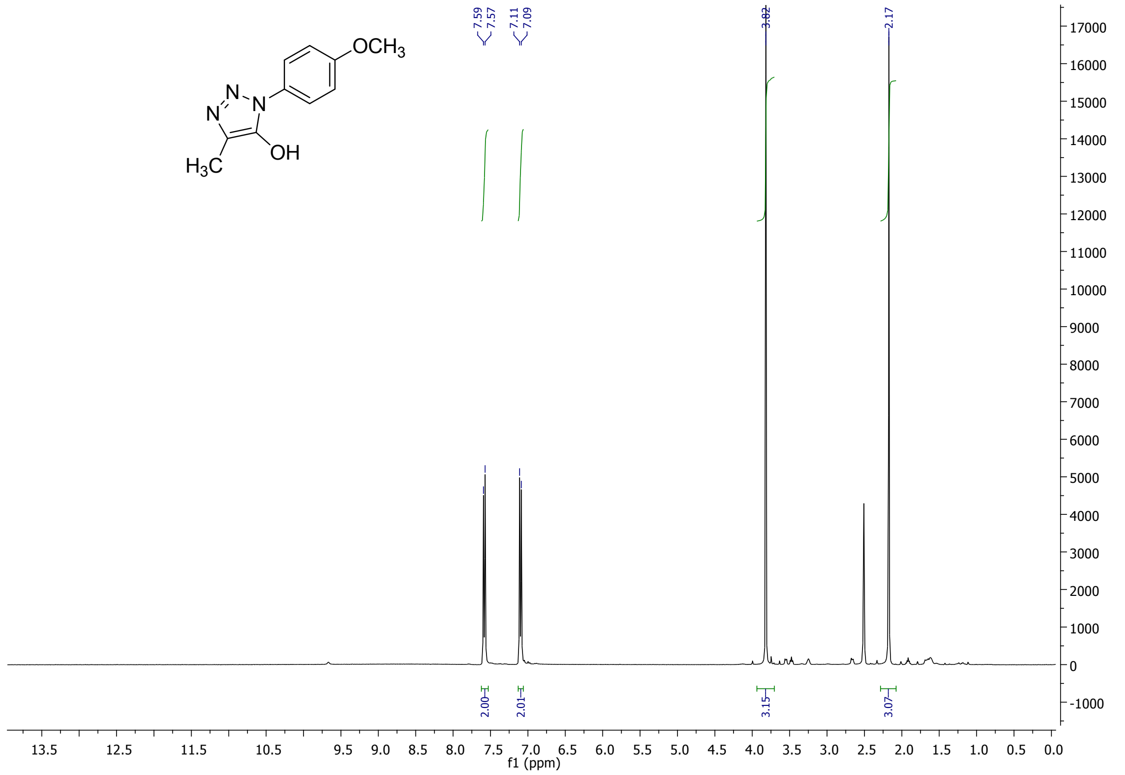

Compound **5c** $^{13}C\{^1H\}$ NMR (101 MHz, DMSO-d$_6$)

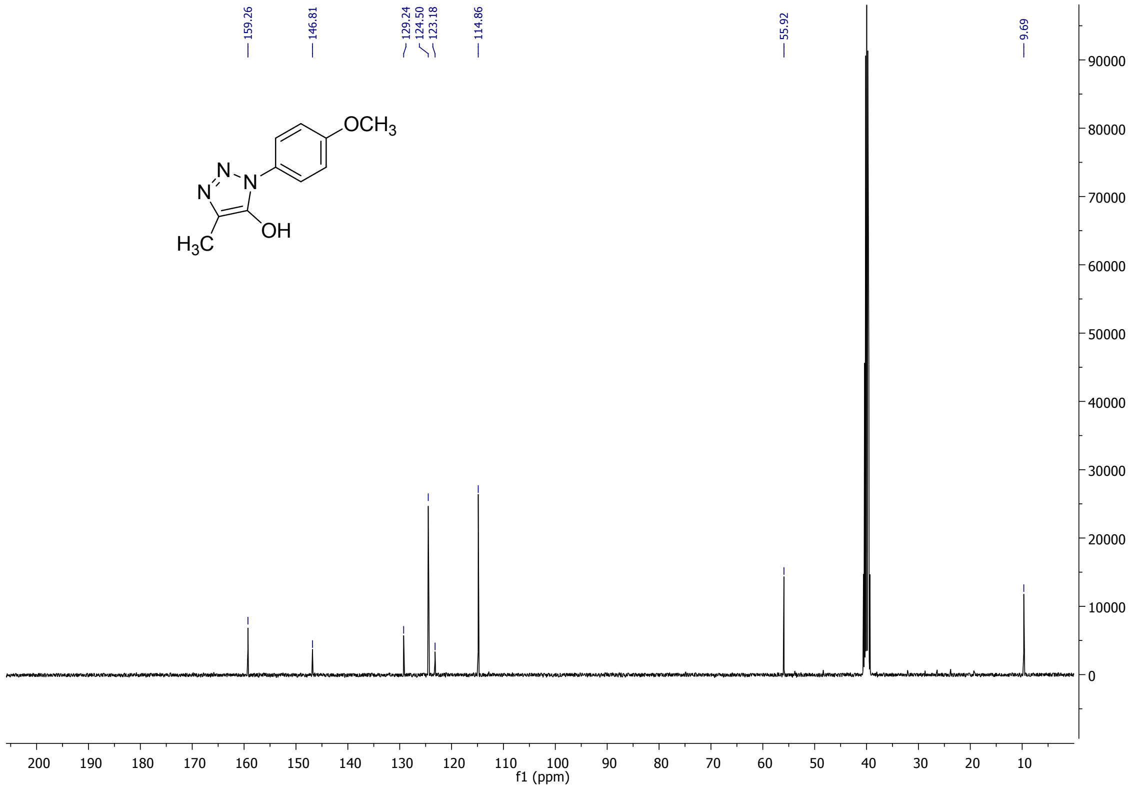

Compound **5d** (400 MHz, DMSO-d_6)

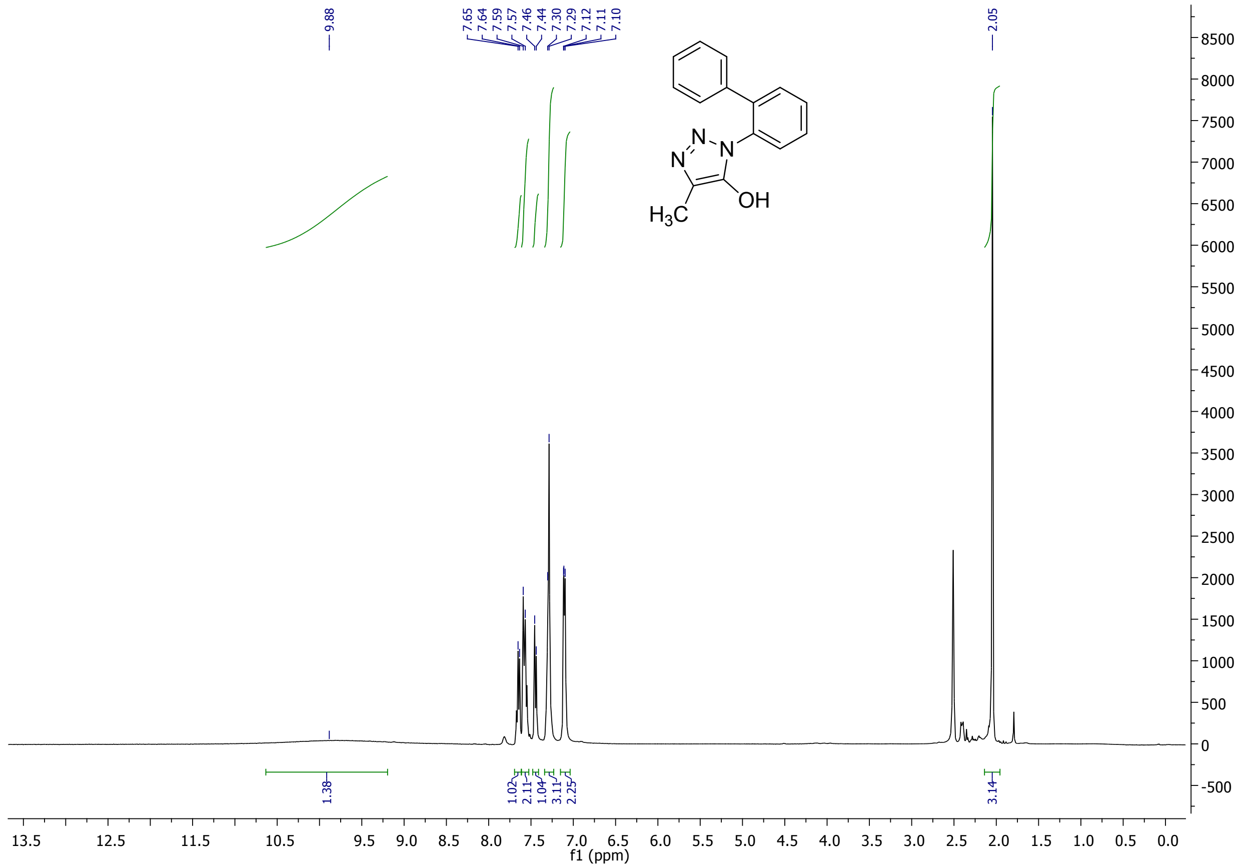

Compound **5d** $^{13}C\{^1H\}$ NMR (101 MHz, DMSO-d_6)

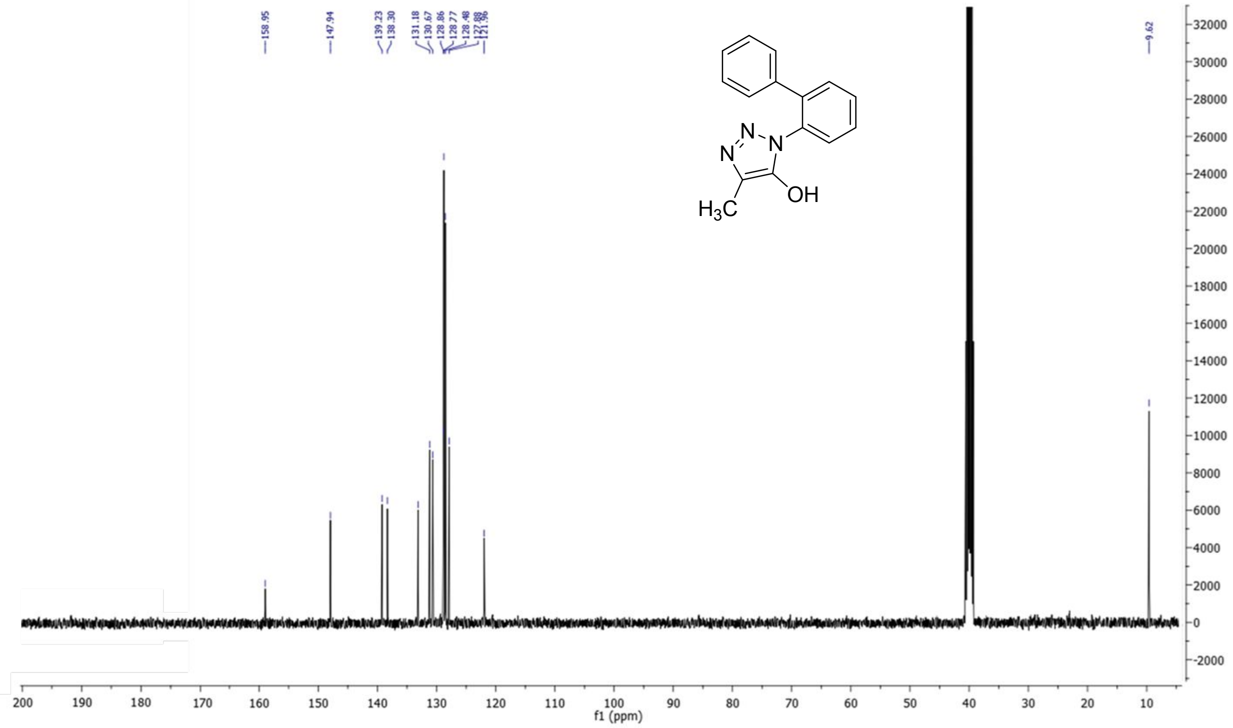

Compound **5e** (400 MHz, DMSO-d$_6$)

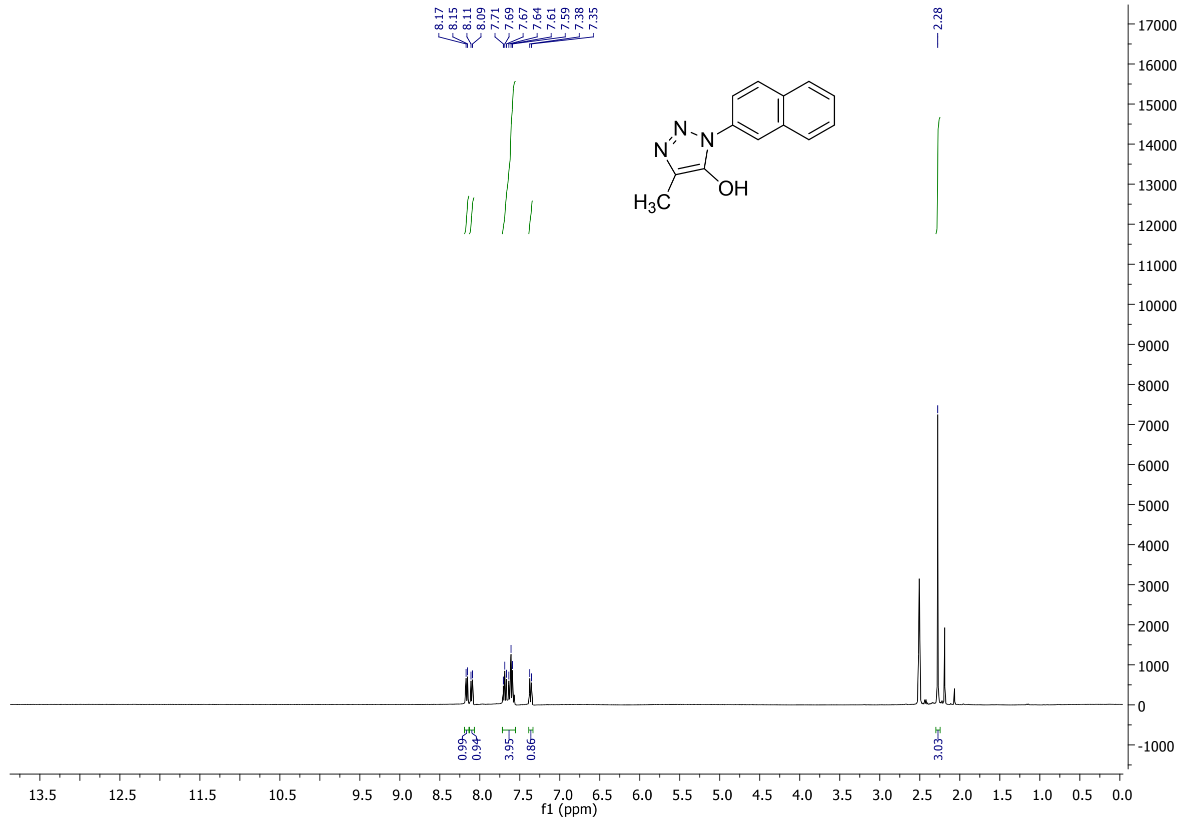

Compound **5e** $^{13}C\{^1H\}$ NMR (101 MHz, DMSO-d$_6$)

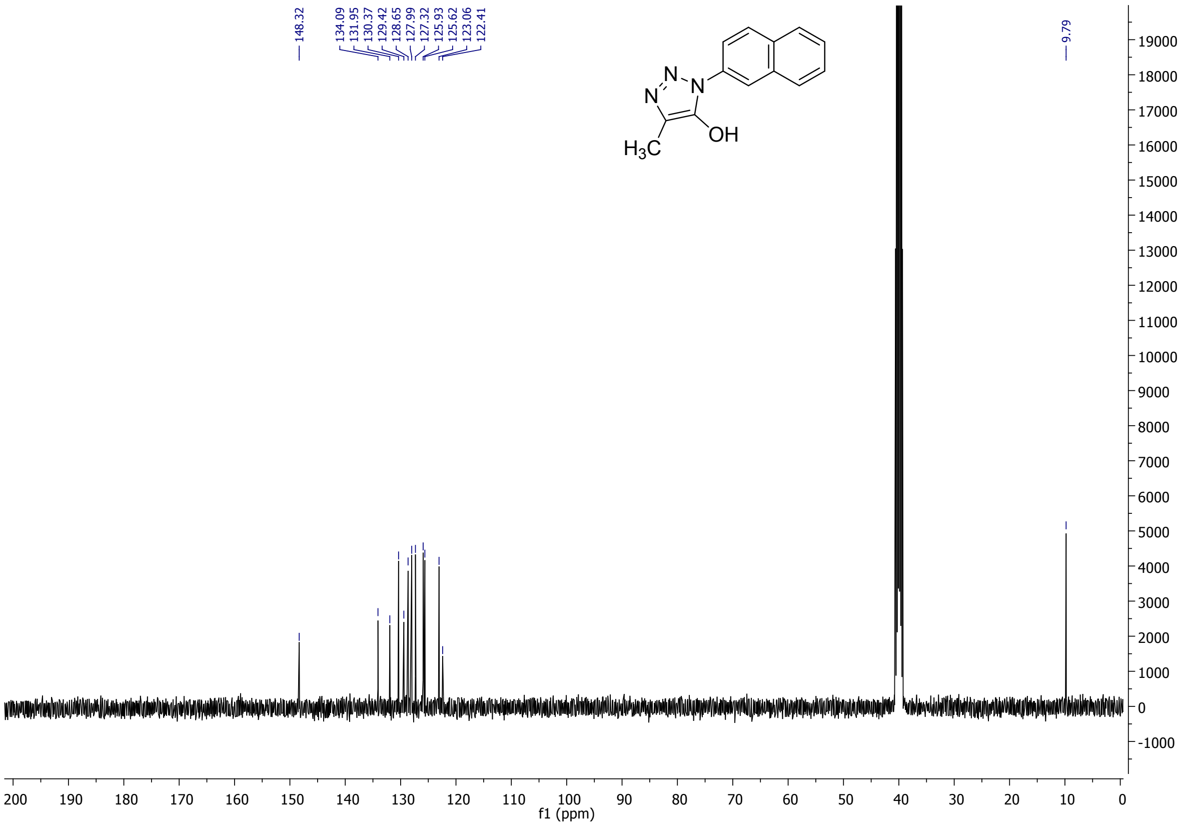

Compound **5f** (400 MHz, DMSO-d$_6$)

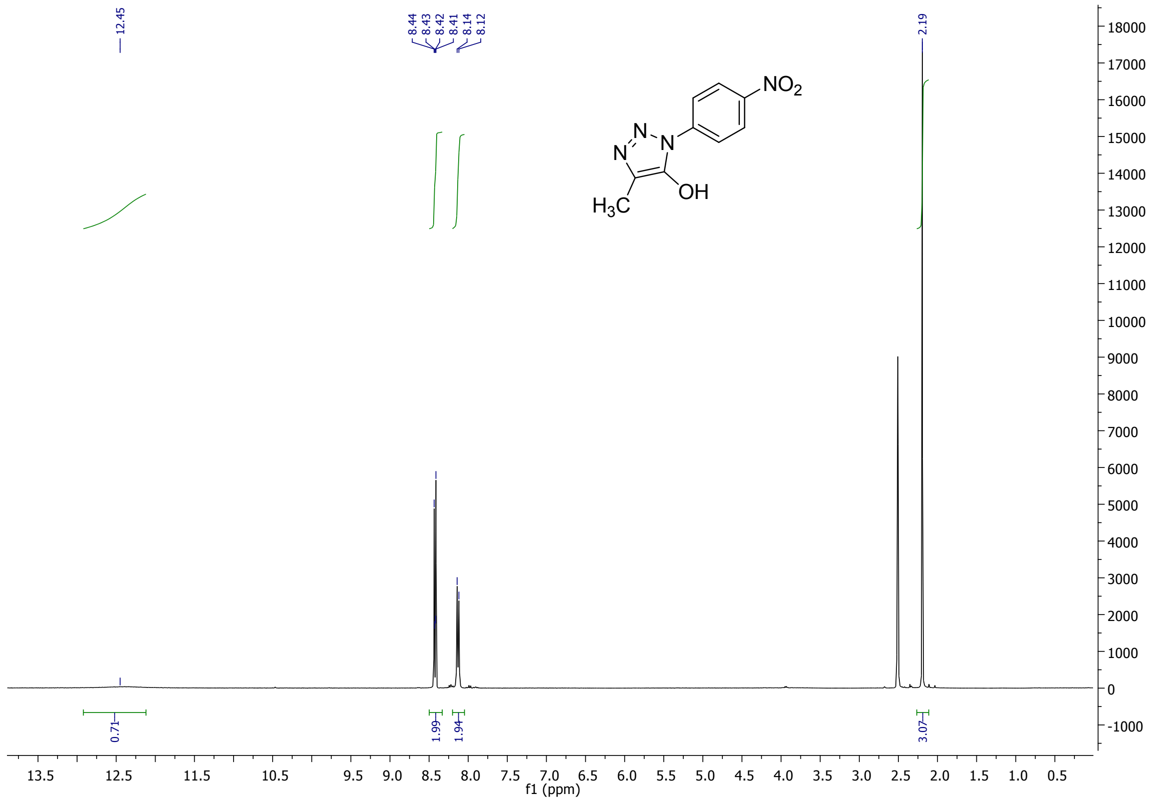

Compound **5f** $^{13}C\{^1H\}$ NMR (101 MHz, DMSO-d$_6$)

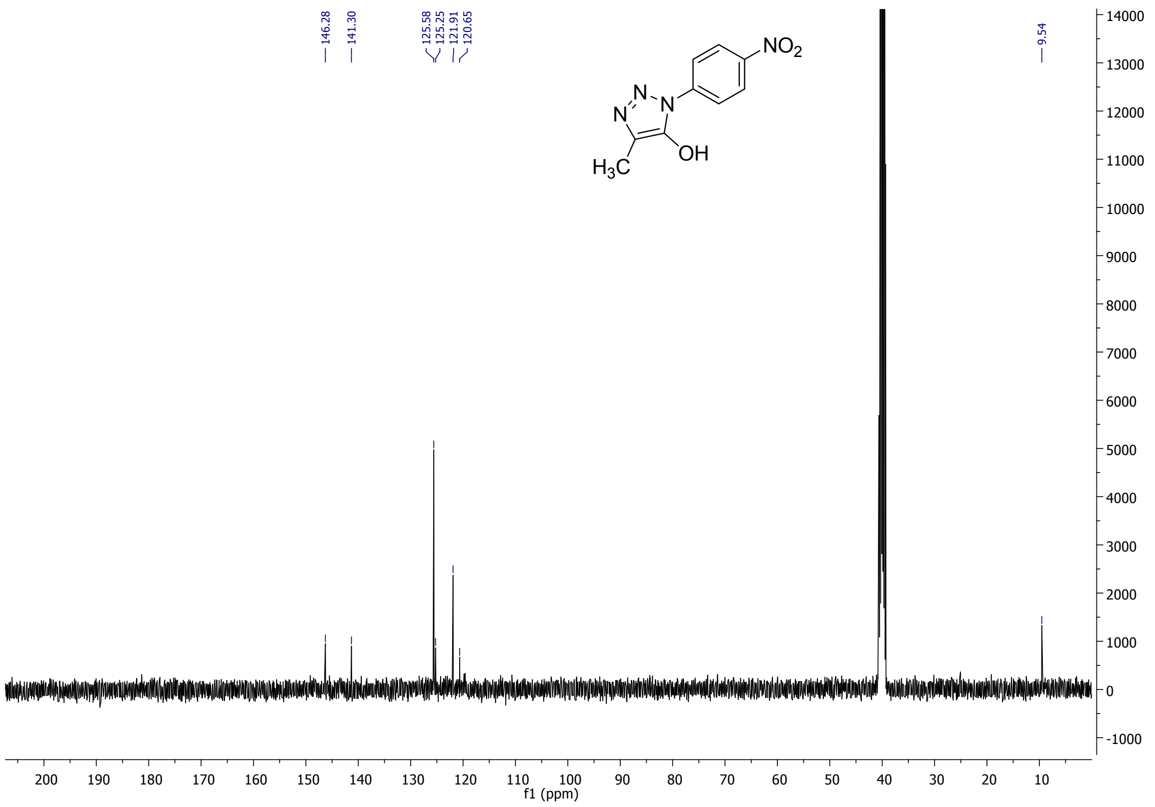

Compound **5g** (400 MHz, CD_3OD)

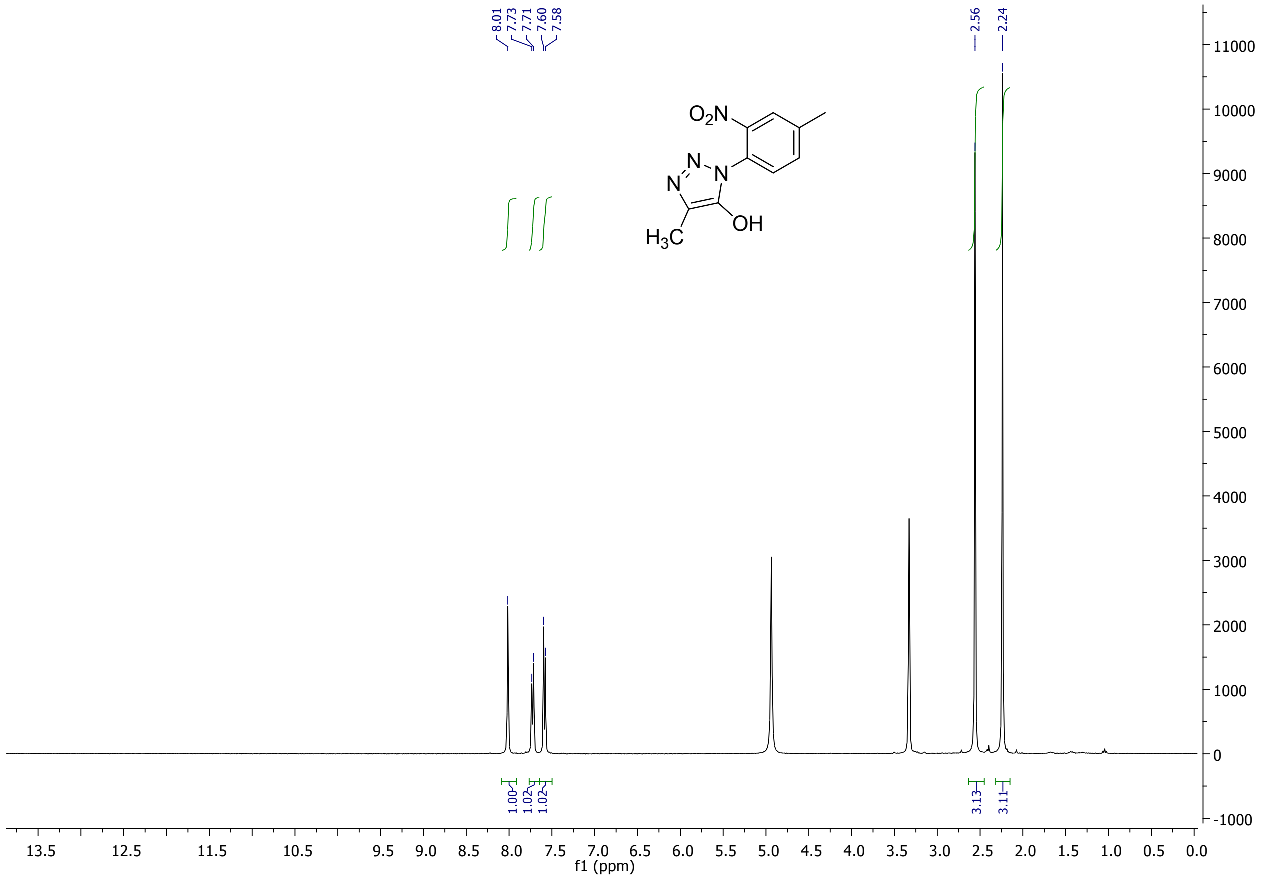

Compound **5g** $^{13}C\{^{1}H\}$ NMR (101 MHz, CD_3OD)

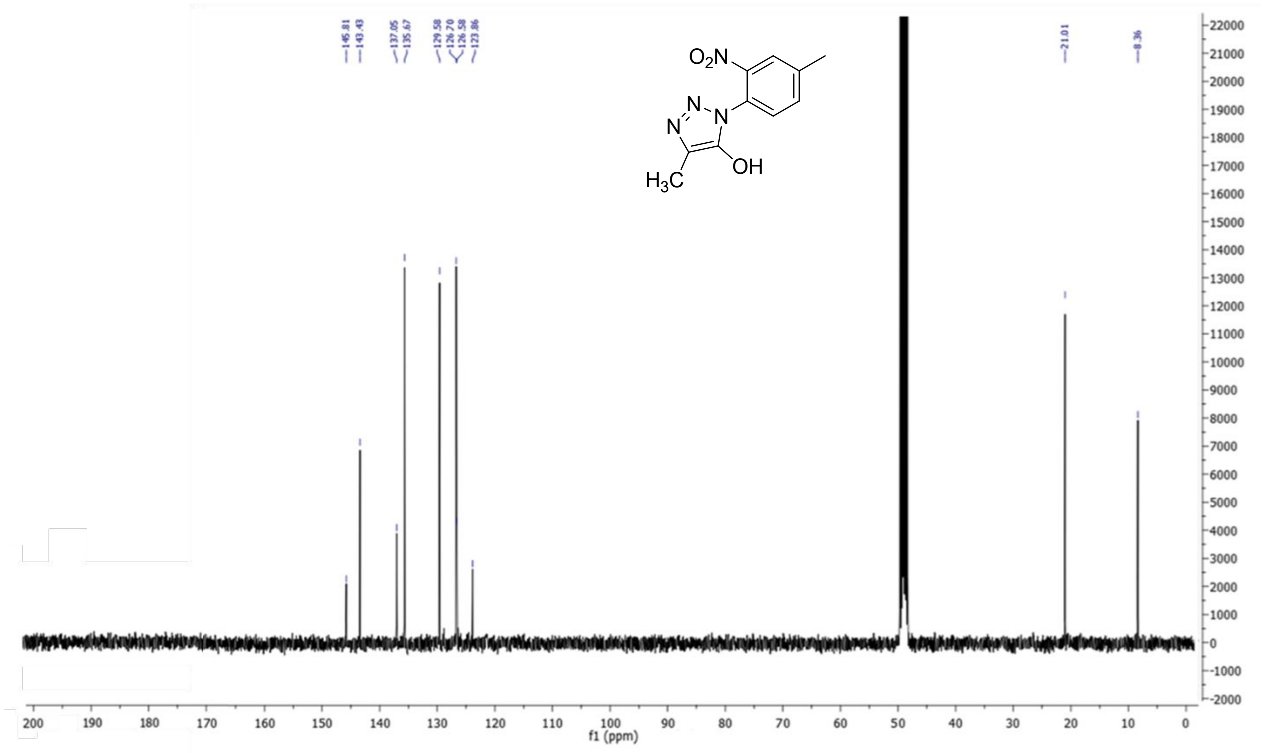

Compound **5k** (400 MHz, DMSO-d$_6$)

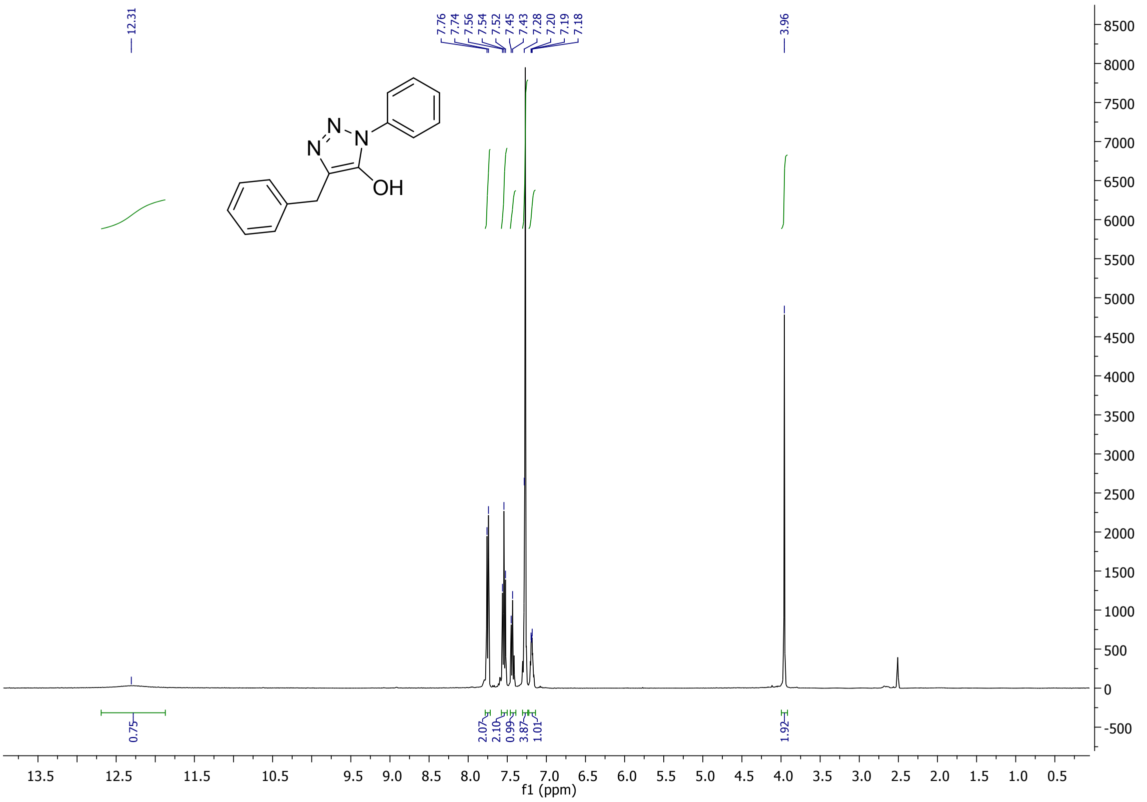

Compound **5k** $^{13}C\{^1H\}$ NMR (101 MHz, DMSO-d$_6$)

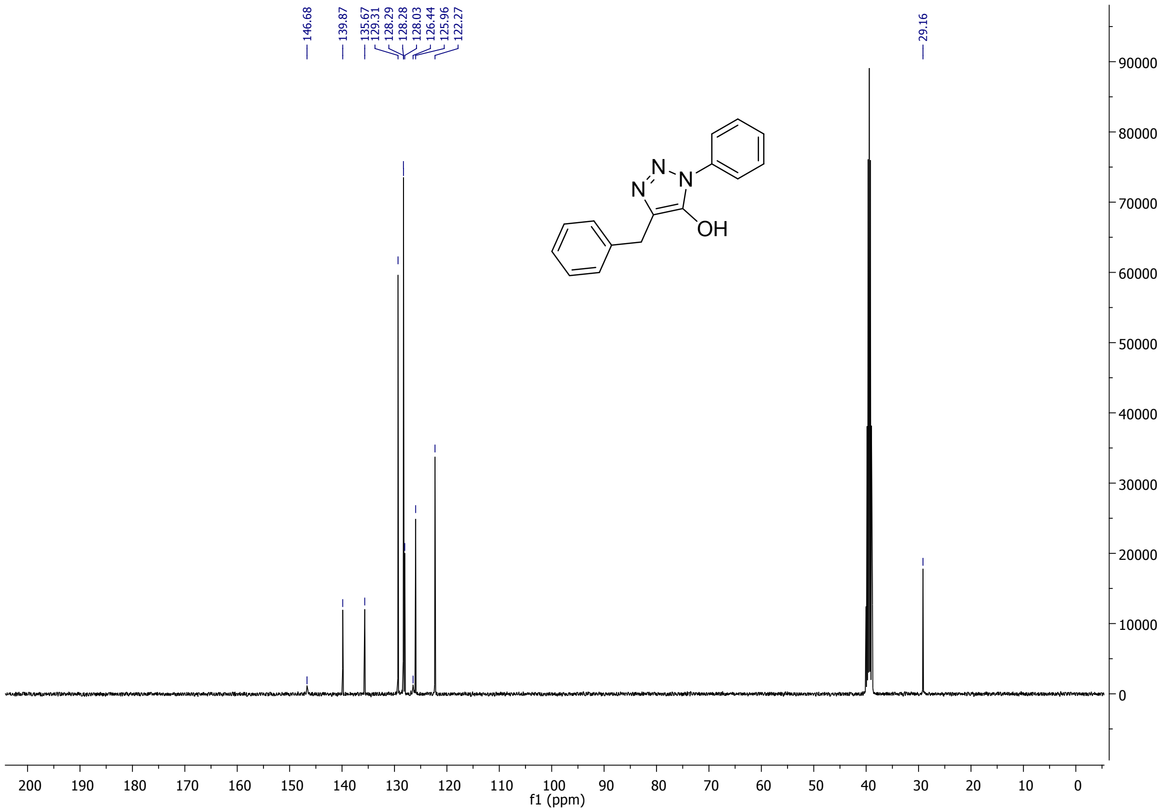

Compound **8a** (400 MHz, $CDCl_3$)

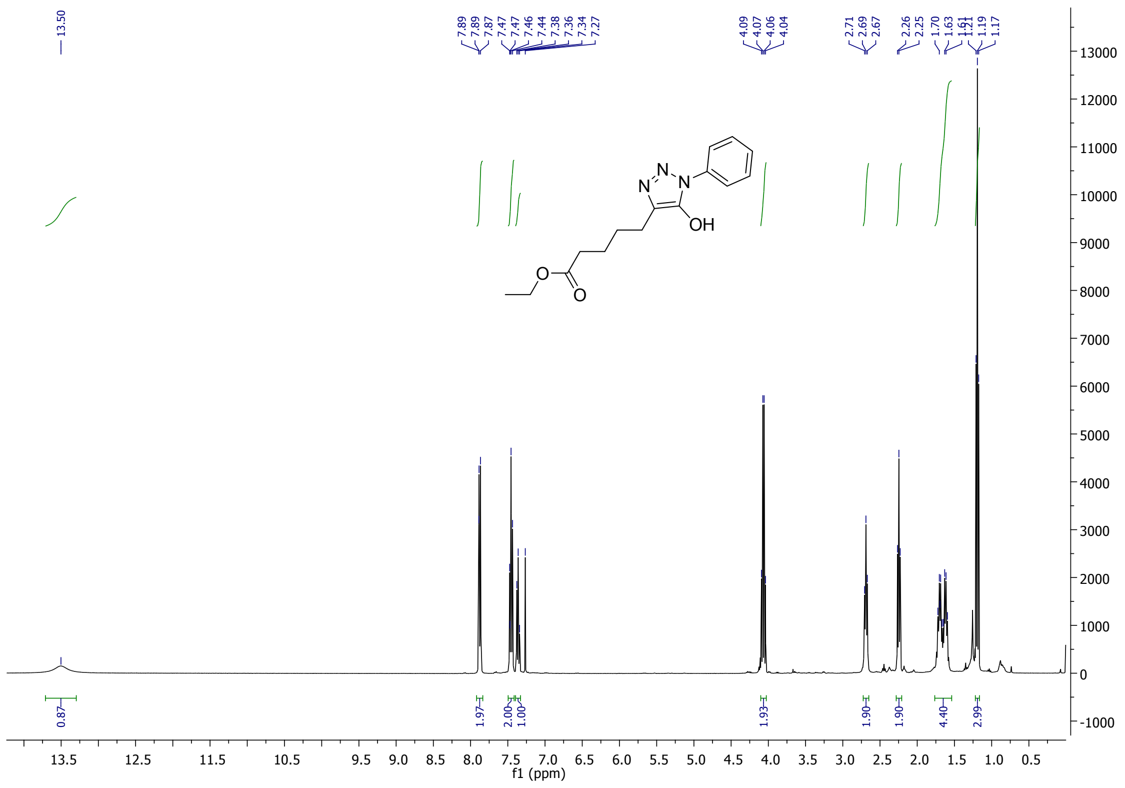

Compound **8a** $^{13}C\{^1H\}$ NMR (101 MHz, $CDCl_3$)

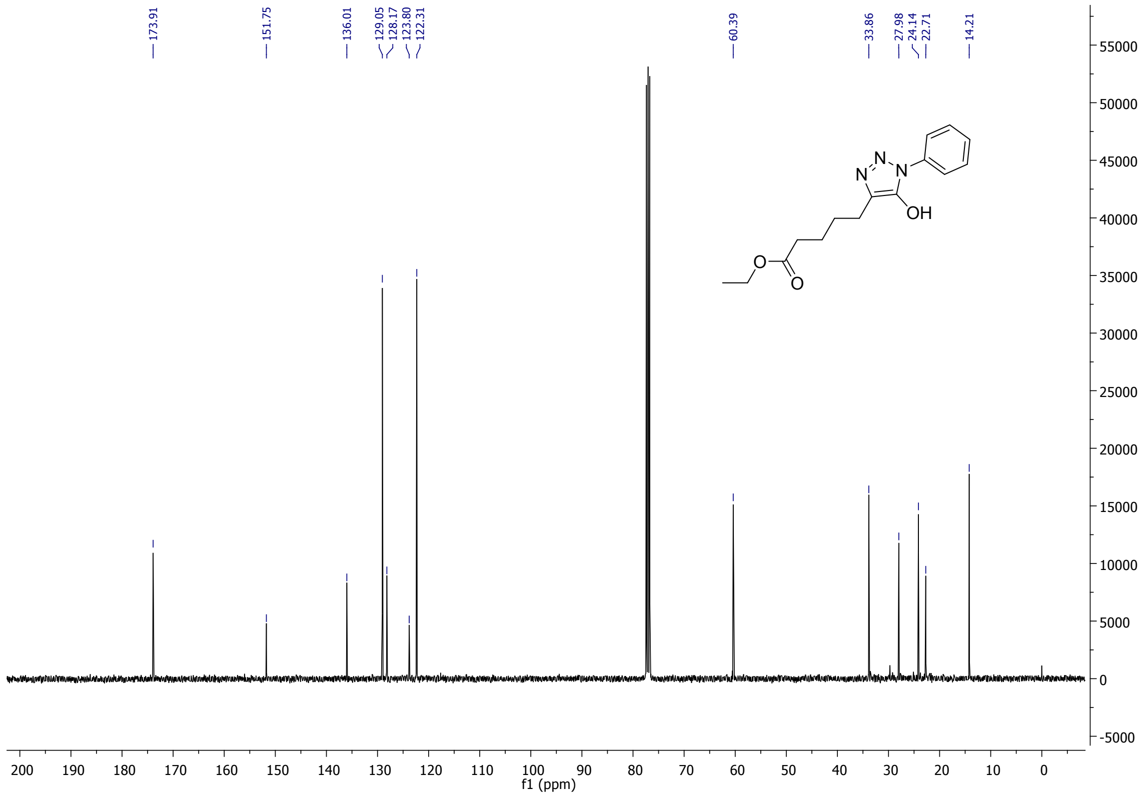

Compound **8b** (400 MHz, $CDCl_3$)

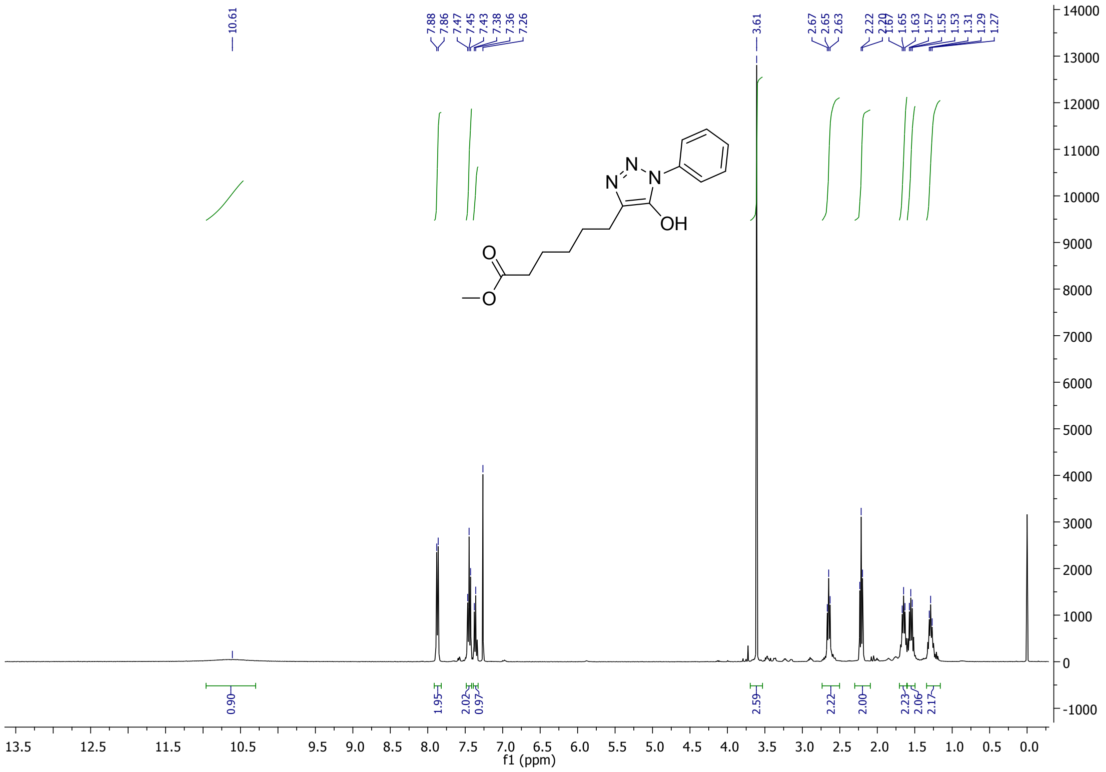

Compound **8b** $^{13}C\{^1H\}$ NMR (101 MHz, $CDCl_3$)

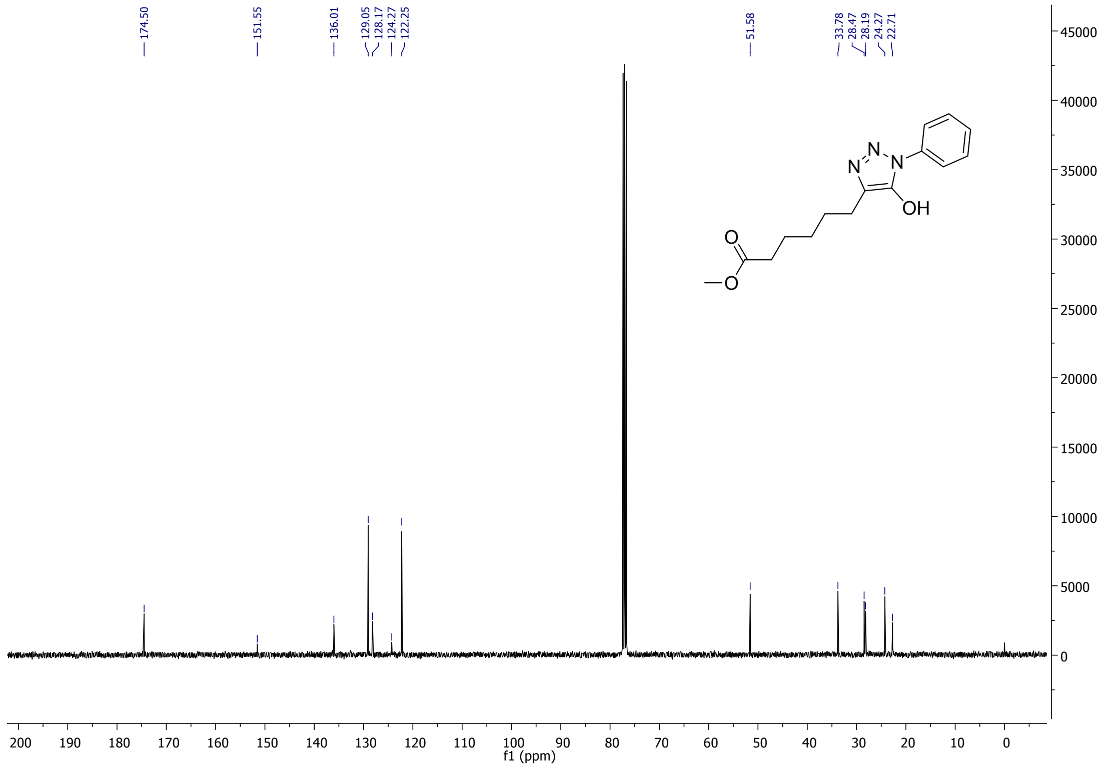

Compound **8c** (400 MHz, $CDCl_3$)

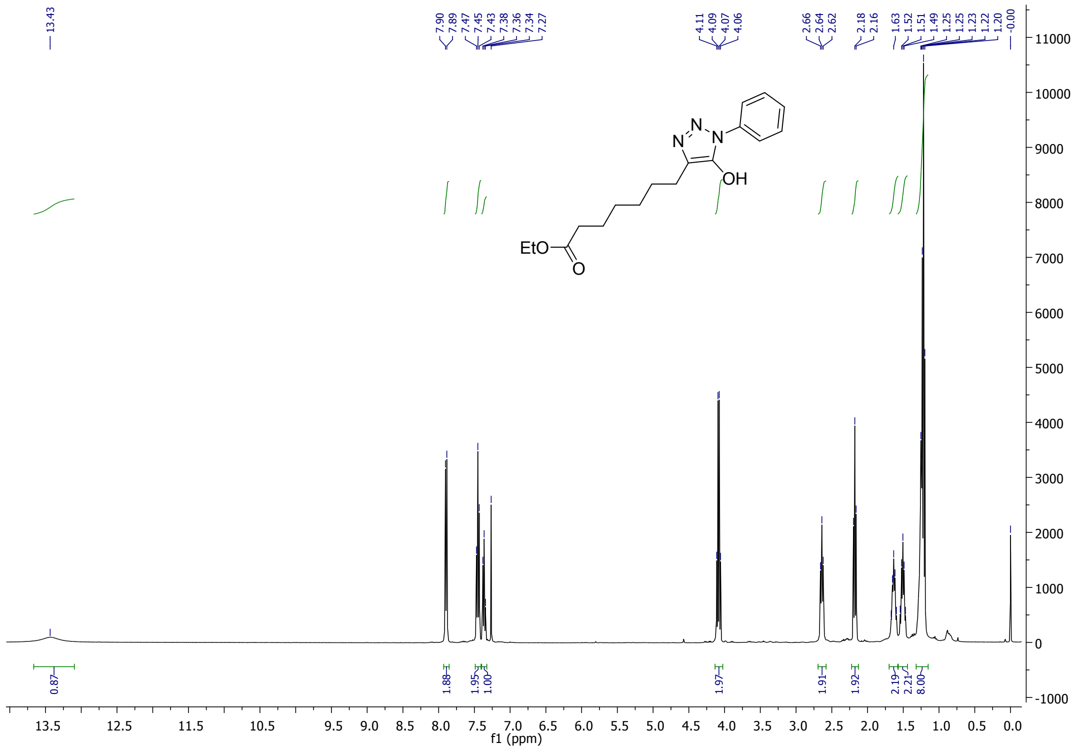

Compound **8c** $^{13}C\{^{1}H\}$ NMR (101 MHz, $CDCl_3$)

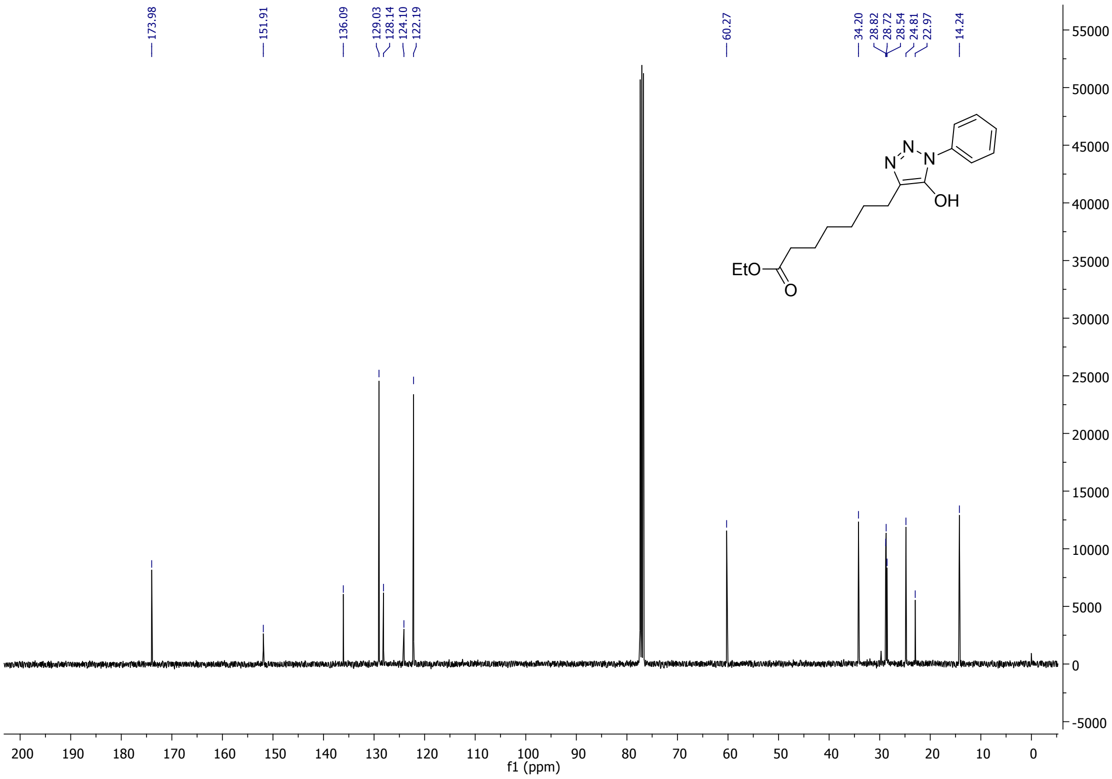

Compound **11a** (400 MHz, DMSO-d$_6$)

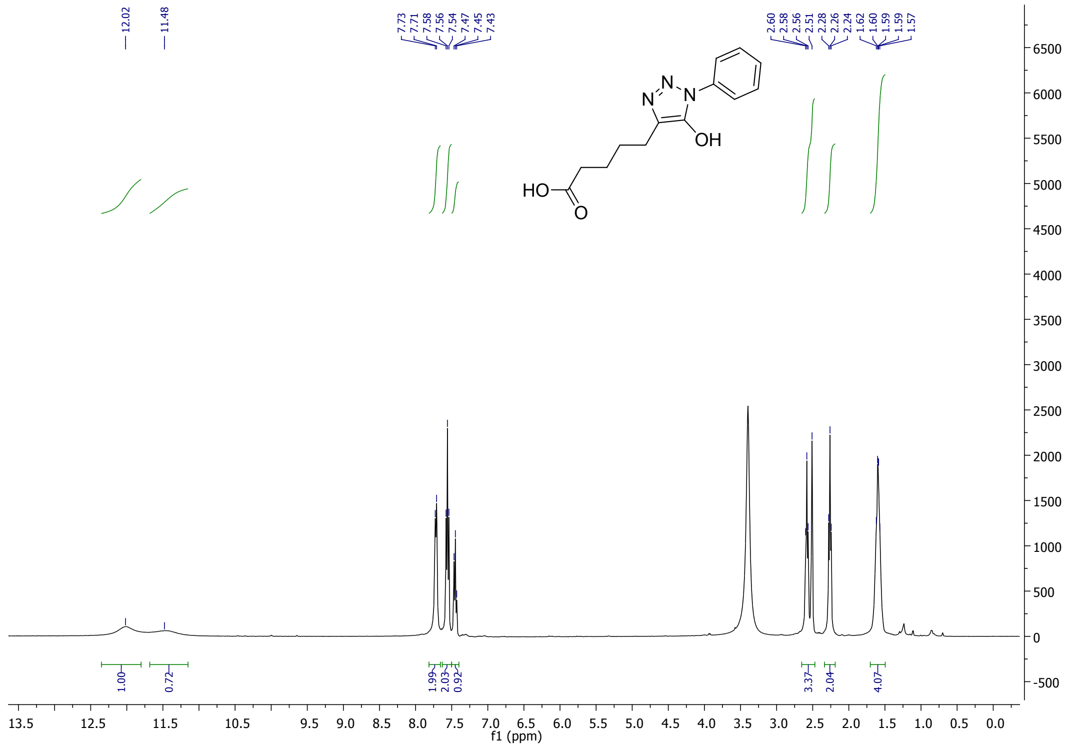

Compound **11a** $^{13}C\{^1H\}$ NMR (101 MHz, DMSO-d$_6$)

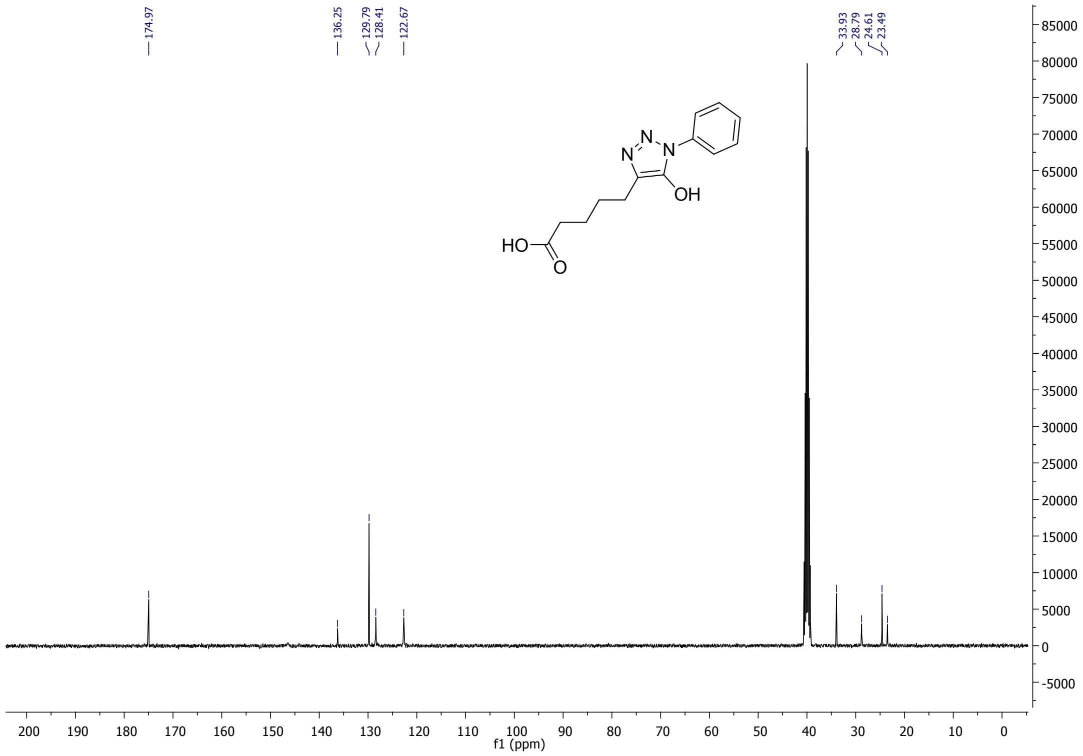

Compound **11b** (400 MHz, DMSO-d$_6$)

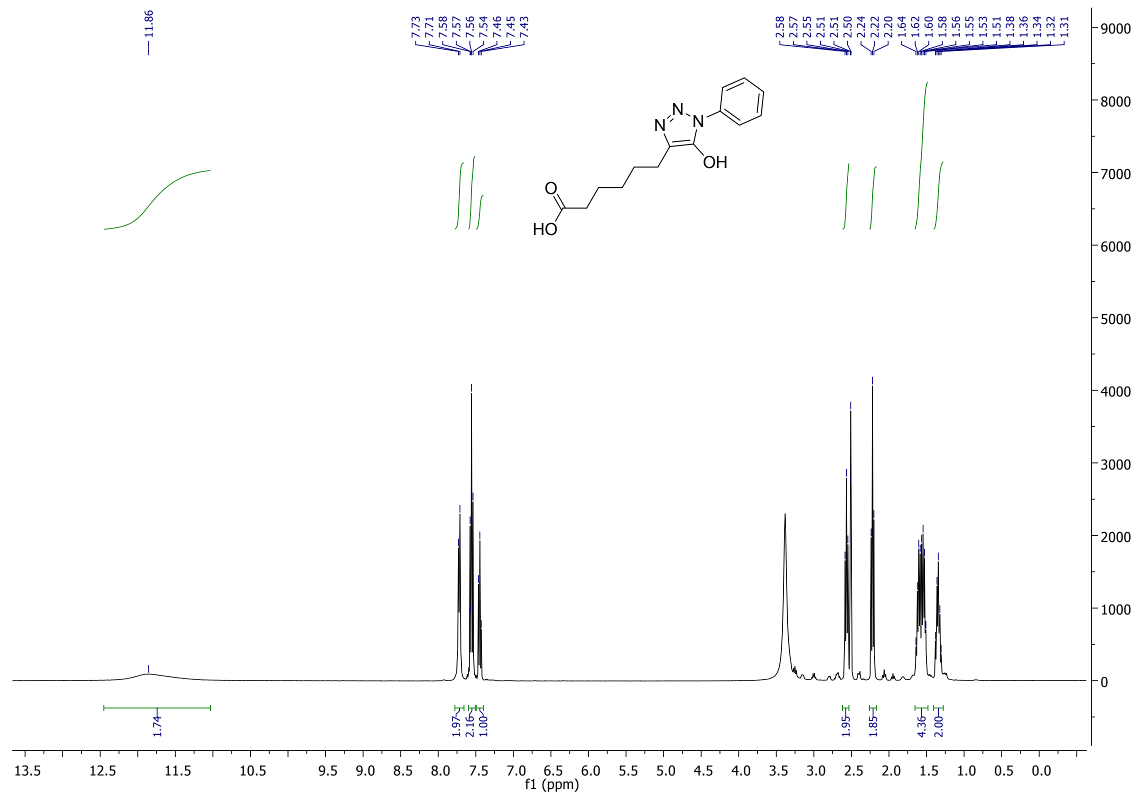

Compound **11b** $^{13}C\{^1H\}$ NMR (101 MHz, DMSO-d$_6$)

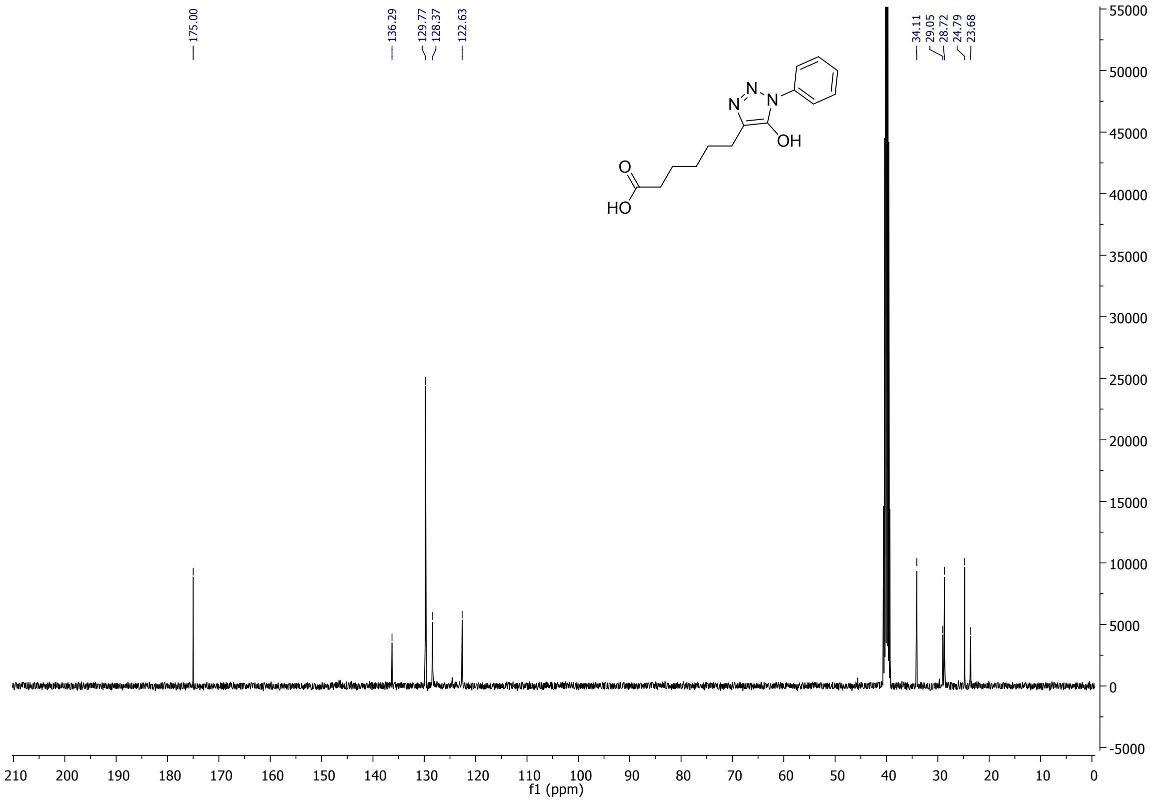

Compound **11c** (400 MHz, DMSO-d$_6$)

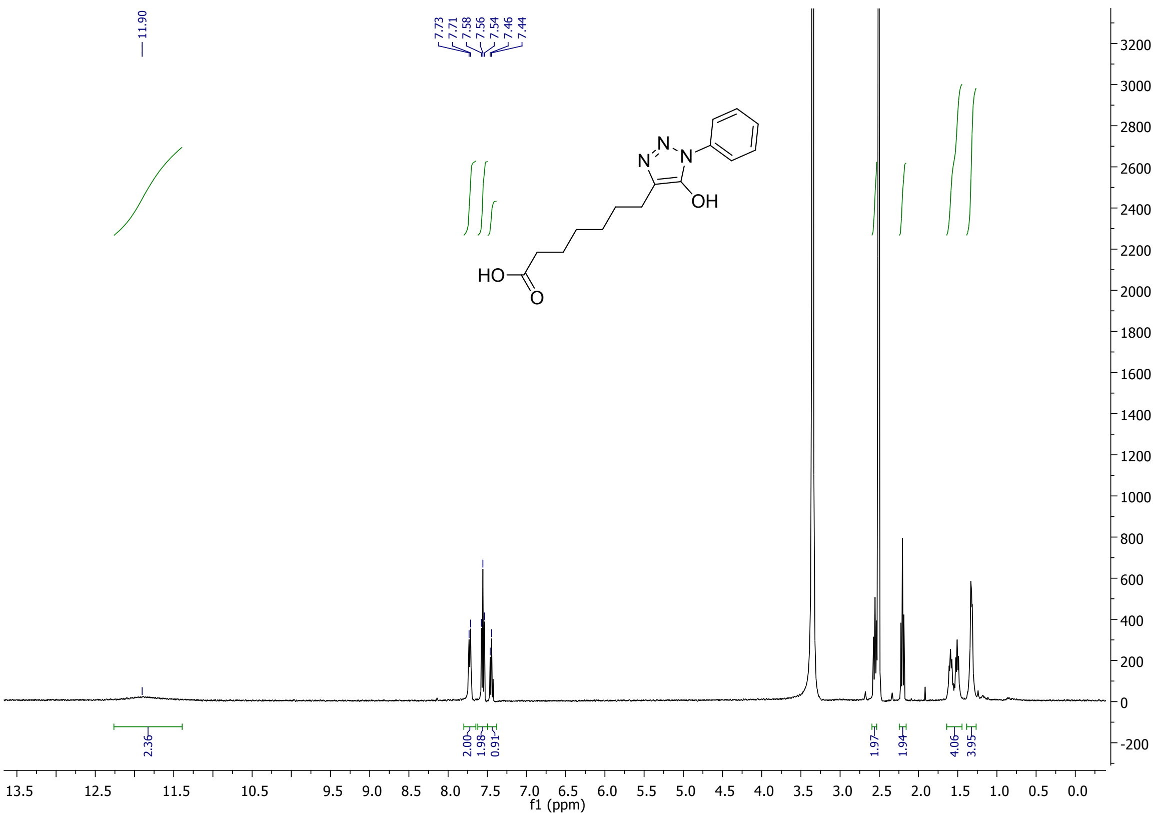

Compound **11c** $^{13}C\{^1H\}$ NMR (101 MHz, DMSO-d$_6$)

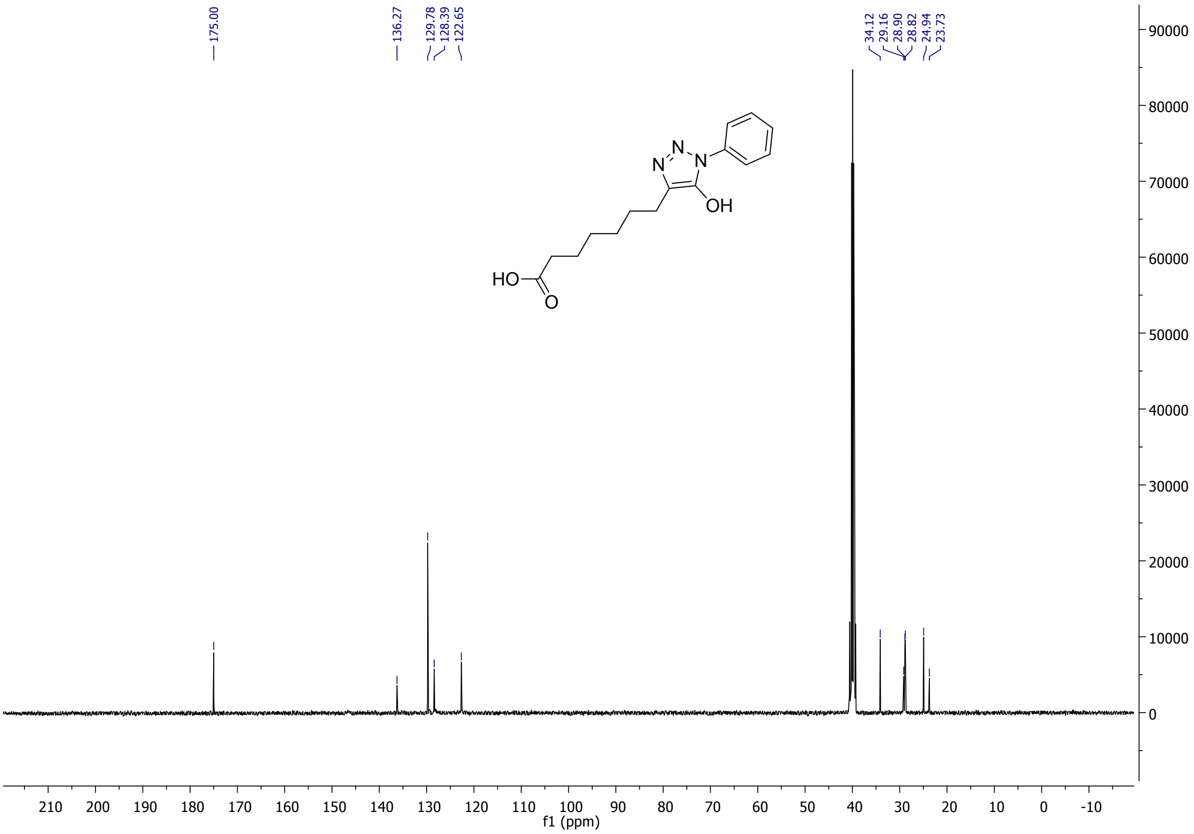

Compound **10a** (400 MHz, DMSO-d_6)

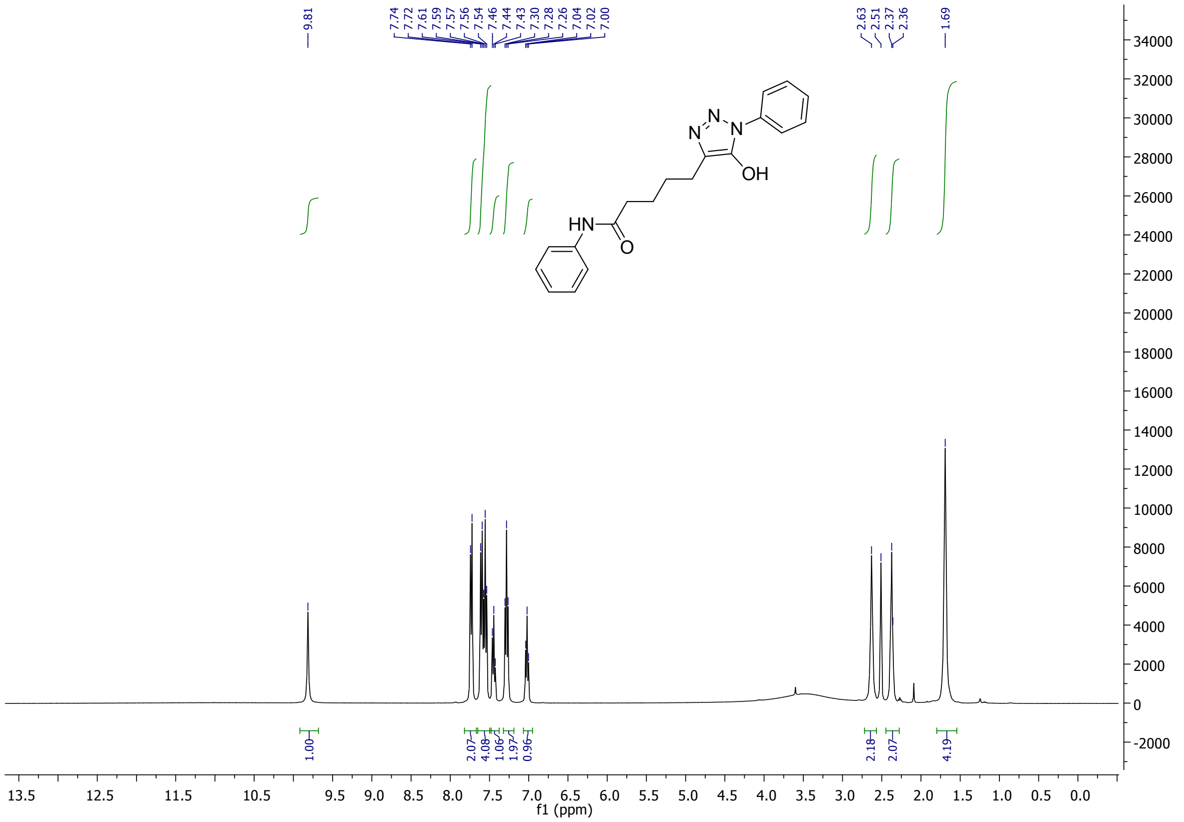

Compound **10a** $^{13}C\{^1H\}$ NMR (101 MHz, DMSO-d_6)

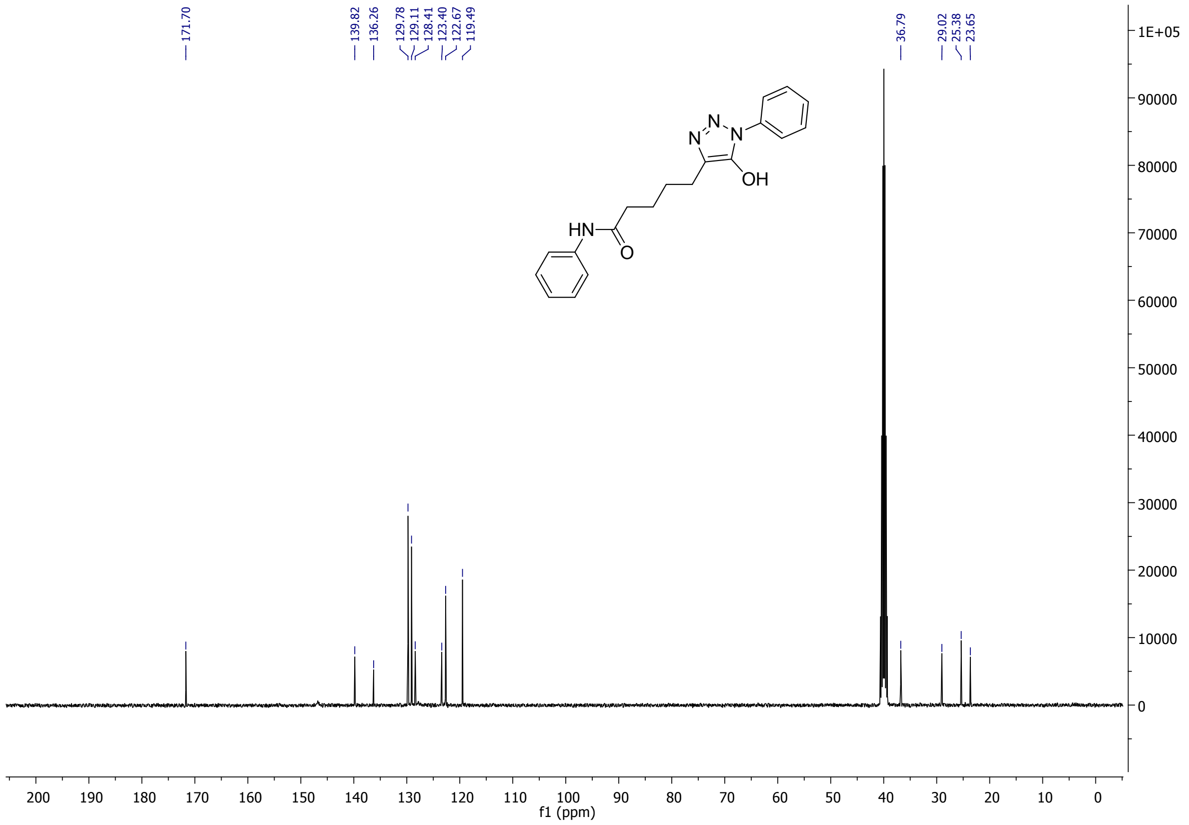

Compound **10b** (400 MHz, DMSO-d_6)

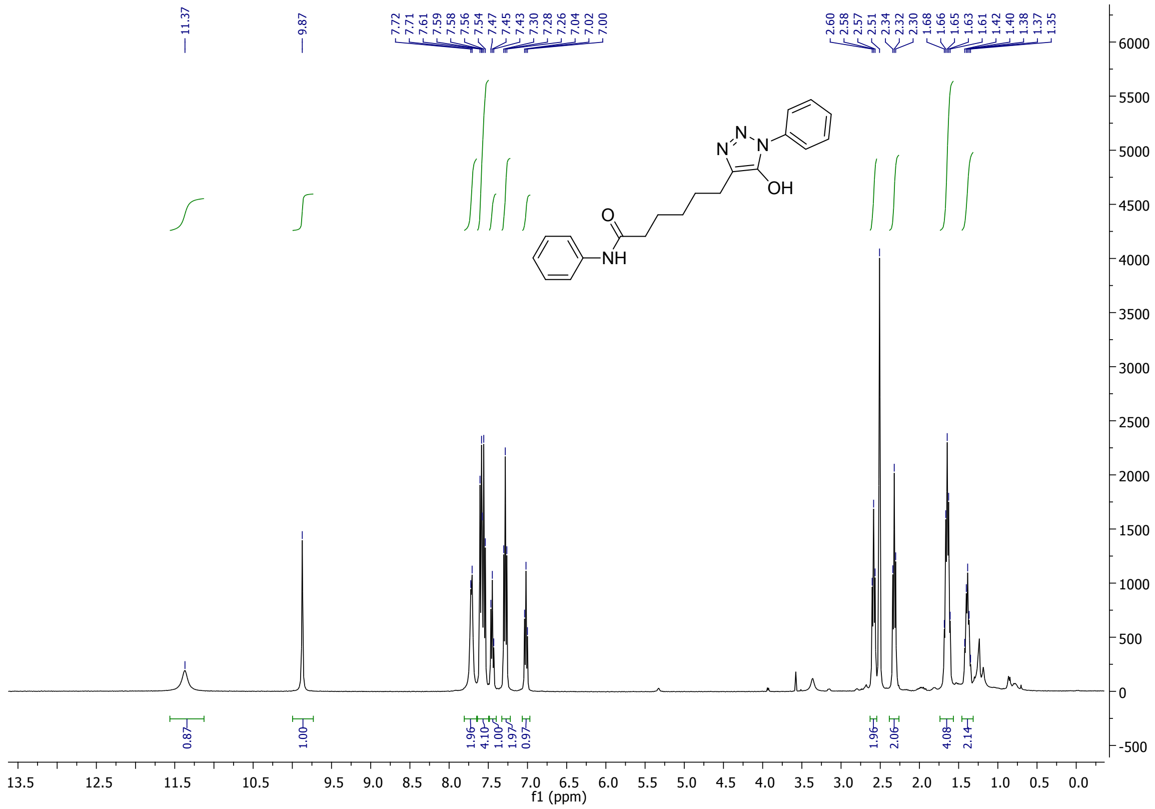

Compound **10b** $^{13}C\{^1H\}$ NMR (101 MHz, DMSO-d_6)

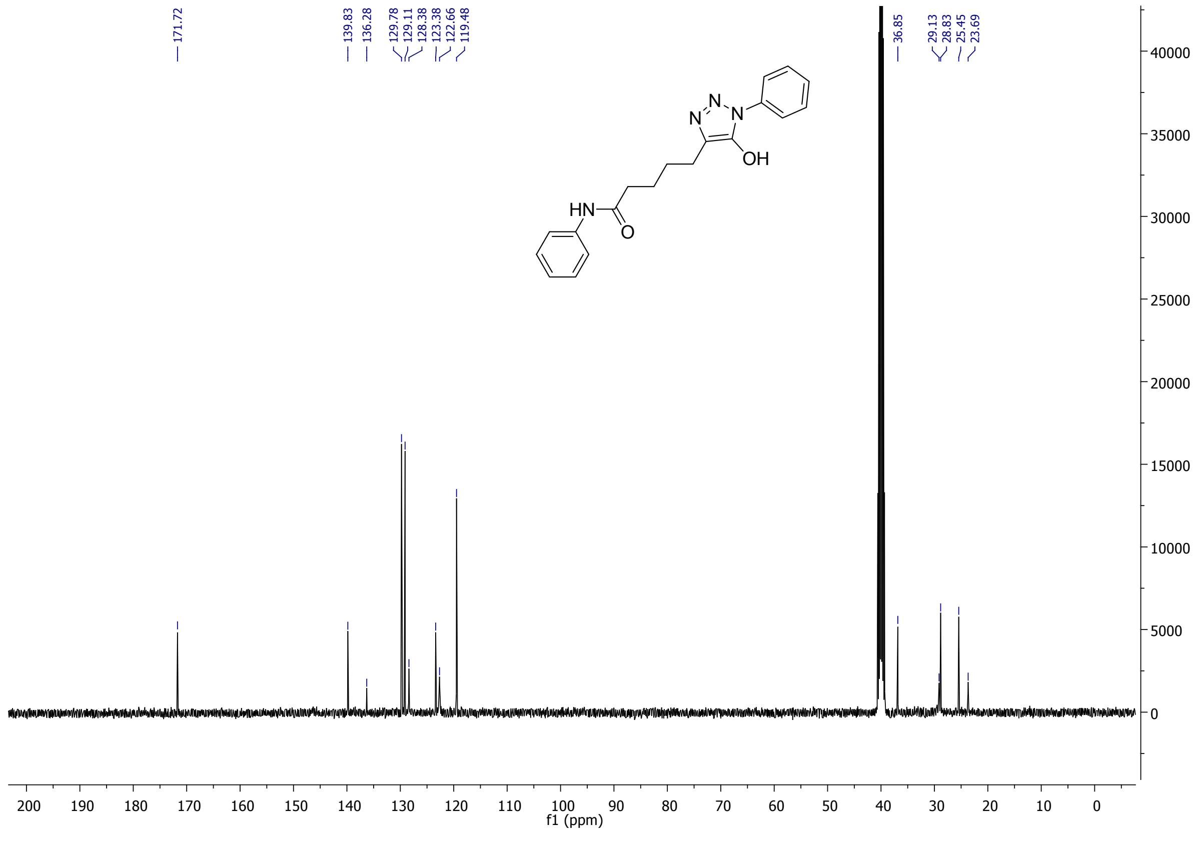

Compound **10c** (400 MHz, DMSO-d$_6$)

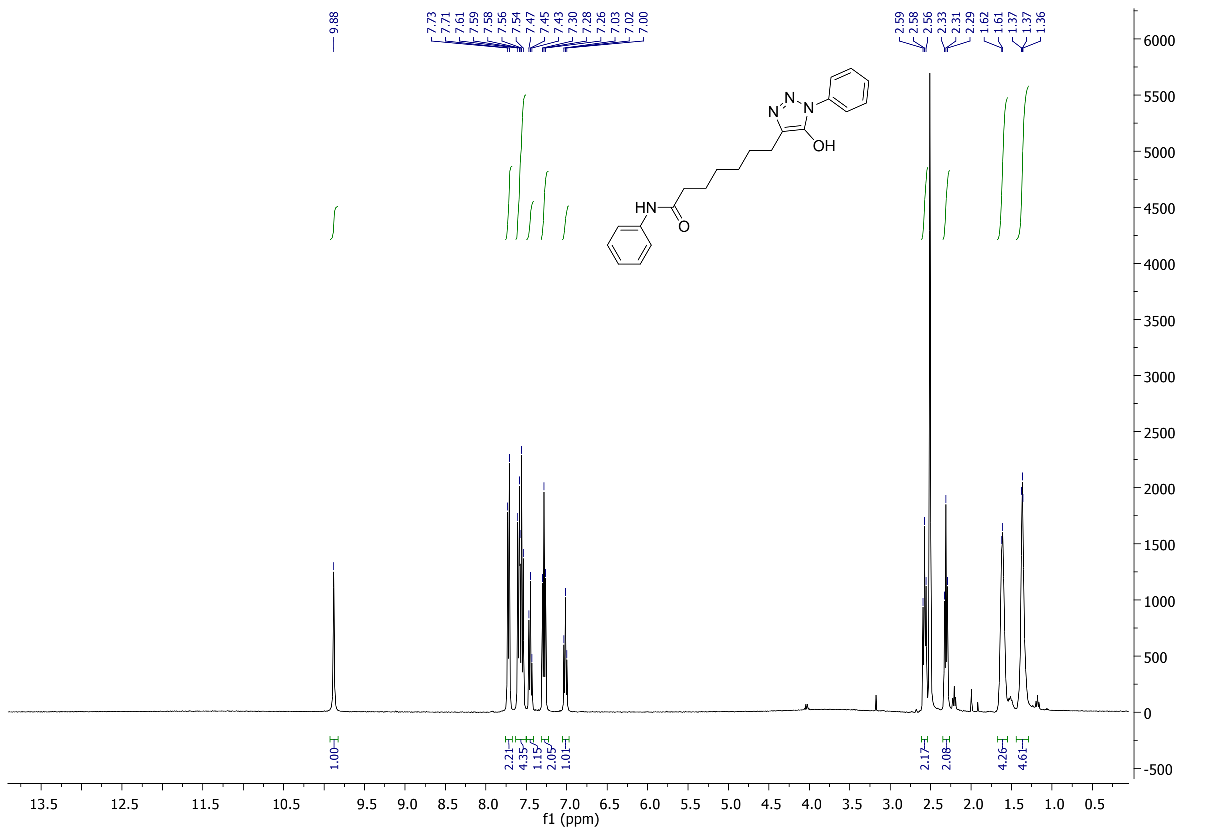

Compound **10c** $^{13}C\{^{1}H\}$ NMR (101 MHz, DMSO-d$_6$)

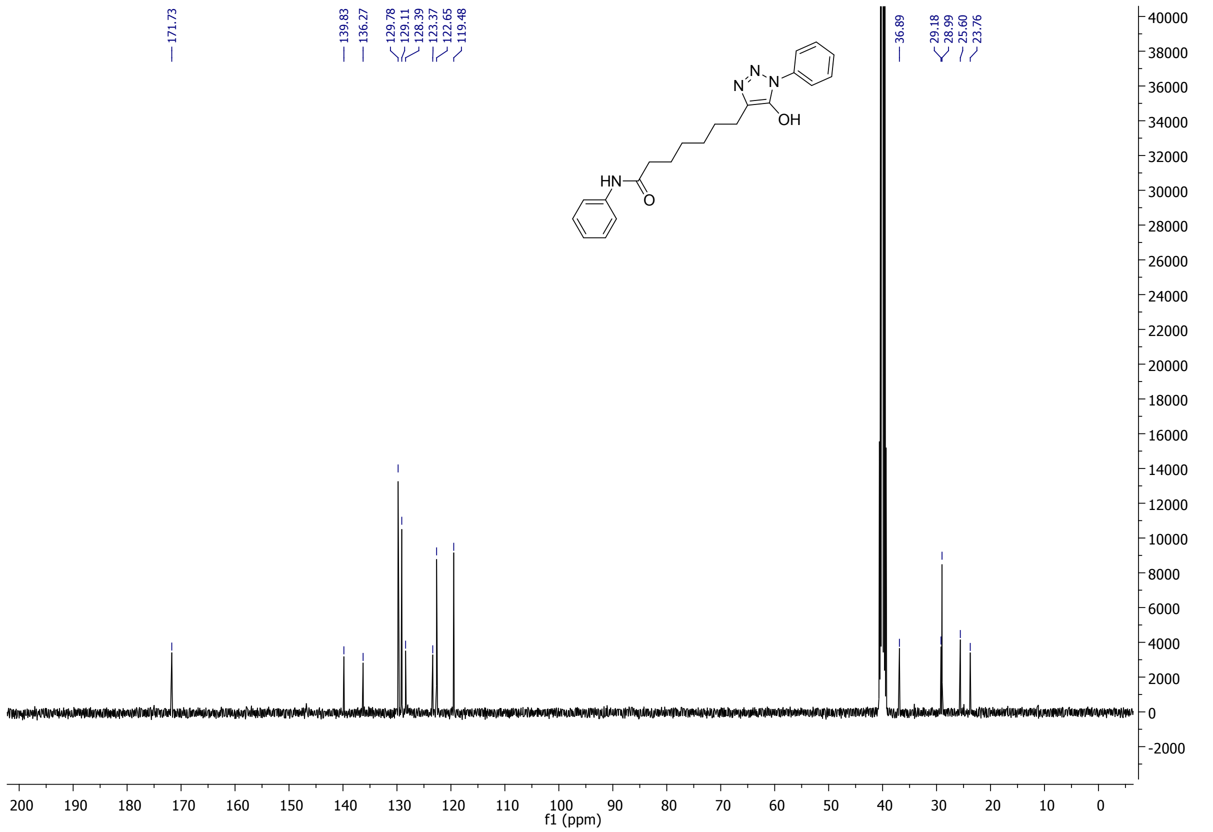

Supplement: Supplementary file 1 — jo1c00778_si_001.pdf [file jo1c00778_si_001.pdf]
